# Antimicrobial properties of hindered amine light stabilizers in polymer coating materials and their mechanism of action

**DOI:** 10.3389/fbioe.2024.1390513

**Published:** 2024-06-24

**Authors:** Tiago Costa, Belém Sampaio-Marques, Nuno M. Neves, Helena Aguilar, Alexandra G. Fraga

**Affiliations:** ^1^ Life and Health Sciences Research Institute (ICVS), School of Medicine, University of Minho, Braga, Portugal; ^2^ ICVS/3B’s—PT Government Associate Laboratory, Braga/Guimarães, Portugal; ^3^ 3B’s Research Group on Biomaterials, Biodegradables and Biomimetics, Headquarters of the European Institute of Excellence on Tissue Engineering and Regenerative Medicine, University of Minho, Guimarães, Portugal; ^4^ Têxtil Manuel Gonçalves—Tecidos Plastificados e Outros Revestimentos Para a Indústria Automóvel, S.A. (TMG Automotive), Largo Comendador Manuel Gonçalves, Guimarães, Portugal

**Keywords:** antimicrobial additives, polymer industry materials, reactive nitrogen species, hindered amine light stabilizer, UV-stabilizers

## Abstract

UV-stabilizers are a class of additives that provide extended polymer resistance to UV-degradation, but have also been suggested to have antimicrobial activity, potentially preventing the spread of pathogens, and inhibiting microbial-induced biodegradation. In this work, we incorporated different UV-stabilizers, a hindered amine light stabilizer (HALS), Tinuvin 770 DF and Tinuvin PA 123, or a hybrid HALS/UV-absorber, Tinuvin 5151, in polyurethane formulations to produce lacquer-films, and tested their antimicrobial activity against *Staphylococcus aureus* (methicillin-resistant and -sensitive strains), *Escherichia coli* and *Candida albicans*. Lacquer-films incorporated with Tinuvin 770 DF showed strong antimicrobial performance against bacteria and fungi, while maintaining cytocompatibility. The mechanism of action revealed a positive relationship between Tinuvin 770 DF concentration, microbial death, and reactive nitrogen species (RNS), suggesting that RNS produced during autoxidation of Tinuvin 770 DF is responsible for the antimicrobial properties of this UV-stabilizer. Conversely, lacquer-films incorporated with Tinuvin 5151 or Tinuvin PA 123 exhibited no antimicrobial properties. Collectively, these results highlight the commercial potential of Tinuvin 770 DF to prevent photo- and biodegradation of polymers, while also inhibiting the spread of potentially harmful pathogens. Furthermore, we provide a better understanding of the mechanism underlying the biocidal activity of HALS associated to autooxidation of the amine group.

## 1 Introduction

Polymers are composed of macromolecules obtained from a large number of covalently linked organic units or monomers (Plummer, 2014; Namazi, 2017). The possibility to functionalize polymers with chemical groups (Makvandi et al., 2021), aligned with a great diversity of natural-, synthetic-, or semi-synthetic sources (Jayasree et al., 2014), has expanded their use worldwide. The application of these materials are widespread across various industries including construction (Shen et al., 2020), packaging (Vallejos et al., 2022), biomedical fields (tissue engineering and drug delivery) (Bolívar-Monsalve et al., 2021), medical devices and implants (Teo et al., 2016), daily products (household cleaning (Pecquet et al., 2019) and cosmetic products (Alves et al., 2020)) as well as in the automotive industry (Vieyra et al., 2022).

Commercial polymers generally incorporate one or more additives, which can include plasticizers, lubricants, ultraviolet (UV) stabilizers, flame retardants, tougheners, or pigments. These additives serve to facilitate polymer processability or to improve specific properties of the final product, like durability, lifespan, chemical and physical resistance, stability and aesthetics (Gu et al., 2007; Candlin et al., 2008; Plummer, 2014; Wang et al., 2021). Exposure to atmospheric gases and UV radiation can accelerate the degradation process of polymer materials, diminishing their commercial value and functionality (Shrivastava, 2018). In the presence of oxygen, UV radiation can be absorbed by the polymeric material and induce the generation of oxygen-functional groups, such as carbonyl (C=O), carboxyl (COOH) and/or peroxide (O–O) groups (Chin, 2007). These reactions can cause the degradation of the polymeric chains through scission, loss of chemical groups, branching, crosslinking and disordered rearrangement (Chin, 2007). The main consequences of UV-exposure include colour changes; loss of surface gloss; mechanical properties deterioration and ultimately cracking due to chain scission (Chin, 2007). To address these challenges, UV-stabilizers have been incorporated as additives in polymeric formulations to mitigate photodegradation of materials and enhance material durability (Nikafshar et al., 2017).

UV-absorbers and hindered amine light stabilizers (HALS) are the most common UV-stabilizer agents used in polymeric materials. UV-absorbers act by absorbing incident light and dissipating it into thermal energy by intermolecular transfer of protons (Shrivastava, 2018). Examples of UV-absorbers include: benzotriazoles, benzophenones, and hydroxyphenyl triazines (Shrivastava, 2018). On the other hand, HALS act by inhibiting the autoxidation process and inducing the formation of nitroxide radicals from its parent amines. These nitroxide radicals stop the oxidative degradation of the material by coupling to alkyl radicals (Gou et al., 2014). Some HALS are commercialized by BASF, under the commercial name of Tinuvin, such as Tinuvin 5151, Tinuvin 326, Tinuvin 328, Tinuvin 1130, Tinuvin PA 123 and Tinuvin 770 DF (Nikafshar et al., 2017). According to the technical data sheet from the supplier, Tinuvin 770 DF has been approved for use in polyurethane (PUR), polyamide (PA), styrene-butadiene-styrene (SBS), styrene-isoprene-styrene (SIS), ethylene-vinyl-acetate (EVA) polymer systems, solvent-based adhesives (such as acrylic and PUR), and sealants (like MS polymer). Tinuvin 5151 is recommended for pigmented polymer materials like thermoplastics and water-borne polymer systems, including acrylics, vinyl, and PUR dispersions. Tinuvin PA 123 can be applied on acrylic/melamine or polyether sulfone (PES)/melamine systems, epoxy/carboxy resins and polyvinyl chloride.

The polymeric materials can also undergo thermal, chemical, or biological degradation, when exposed to biotic and abiotic environmental factors such as heat, chemical pollutants or even microorganisms. Particularly in biodegradation, microorganisms can break down organic material into oligomers, through enzymatic reactions such as hydrolysis and/or oxidation (Barh et al., 2019; He et al., 2021). In the last years, biodegradation of polymeric materials has garnered attention due to the possibility of decreasing microplastic accumulation in the ecosystems (Lee et al., 2023). In fact, due to their composition, based on carbon and nitrogen, polymers are a potential substrate for heterotrophic microorganisms (Katsikogianni et al., 2004; Gu, 2007; Shan, 2022). Specifically, it has been described that certain bacterial and fungal species, including *Staphylococci*, *Pseudomonas*, *Corynebacterium*, *Curvularia senegalensis*, *Aspergillus spp* and *Cladosporium spp*, have been found capable of biodegrading PUR (Howard et al., 2002; Ru et al., 2020). While this presents a potential benefit in terms of circular economy and carbon neutrality, it is important to acknowledge that biological degradation can also contribute to decreased structural integrity, functionality, and commercial value of the polymer (Shilpa et al., 2022). To mitigate this risk, antimicrobial coatings have been developed to avoid the attachment and growth of microorganisms on polymers (Echeverria et al., 2020). These coatings have diverse applications, including medical devices (Polívková et al., 2017; Zeng et al., 2018), food packaging (Dutta et al., 2009), textile industry (Morais et al., 2016), automobile coatings (Stephenson et al., 2014), and marine anti-biofouling (Yang W. J. et al., 2014).

In addition to their primary function, some UV-stabilizers, including HALS based compounds, have been described as having both antioxidant and antimicrobial properties (Samper et al., 2012; Paine et al., 2016; Olczyk et al., 2020; Abualnaja et al., 2021). Some examples of antimicrobial UV-light stabilizers include metal-based compounds, such as titanium dioxide (TiO_2_) (Shang C. et al., 2022), and zinc oxide (ZnO) (Jin et al., 2021), as well as non-metal compounds, like (3,5-benzamide-2,4-dihydroxyphenyl)(phenyl) methanone (UV-CB) (Shan, 2022). In these cases, the antimicrobial effect of the UV-absorbers is attributed to their photocatalytic activity, which leads to the release of metal ions and reactive oxygen species (ROS). These reactive species disrupt cell membranes and the cell membrane transport system, leading to the inactivation of intracellular enzymes and the leakage of cellular content (Shang et al., 2022). Despite this evidence, reports about the specific biological properties of HALS are limited. The available literature is mostly based on modified HALS, generally combined with other active molecules (Paine et al., 2016; Abualnaja et al., 2021). Considering the importance of HALS as an additive to protect the polymer material against UV-degradation and their association to antimicrobial activity, we proposed to definitively elucidate the antimicrobial properties of commercial HALS - Tinuvin 770 DF, Tinuvin PA 123 or the hybrid HALS/UV-absorber Tinuvin 5151 - incorporated in coating materials, particularly in lacquer-films, and explore their mechanism of action.

## 2 Materials and methods

### 2.1 Production of PUR lacquer-films

**FIGURE 1:**
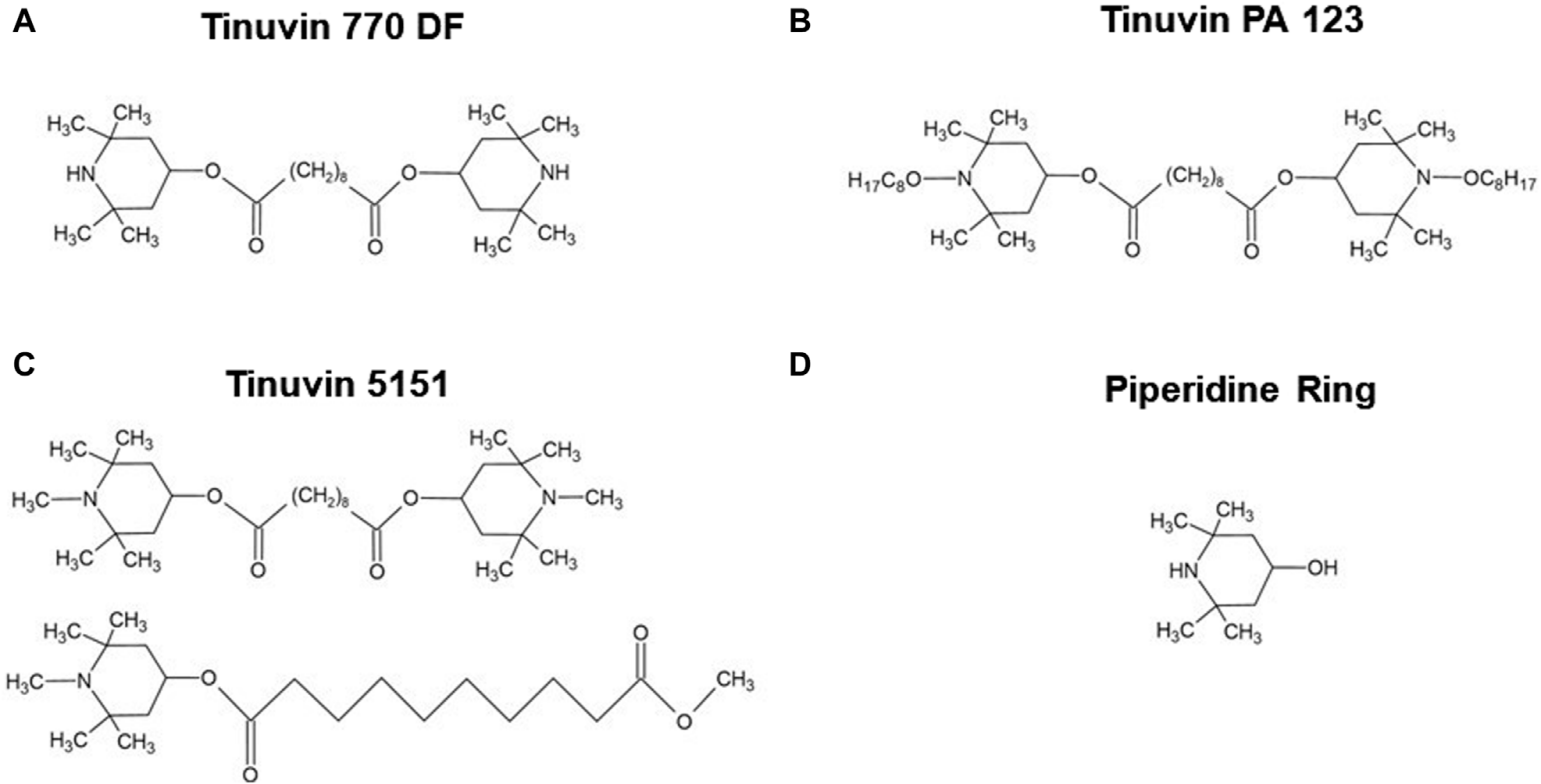
Chemical structure of Hindered Amine Light Stabilizers. **(A)** Tinuvin 770 DF, bis(2,2,6,6-tetramethyl-4-piperidyl) sebacate; **(B)** Tinuvin PA 123, bis(1-octyloxy-2,2,6,6-tetramethyl-4-piperidyl)sebacate; **(C)** Tinuvin 5151 composed by hydrophilic 2-(2-hydroxyphenyl)-benzotriazole as UV absorber and a basic HALS; and **(D)** piperidine ring, 4-hydroxy-2,2,6,6-tetramethylpiperidine. Drawn in ACD/ChemSketch (Freeware) 2022.2.

The base lacquer-formulations were produced according to the UV-stabilizer specifications and solvent system provided by the suppliers. The selected UV-stabilizers include: (i) a HALS, bis(2,2,6,6-tetramethyl-4-piperidyl) sebacate, C_28_H_52_O_4_N_2_, CAS no. 52829-07-9 with the commercial name of Tinuvin 770 DF; (ii) a HALS, bis(1-octyloxy-2,2,6,6-tetramethyl-4-piperidyl)sebacate, C_44_H_84_N_2_O_6_, CAS no. 129757-67-1 with the commercial name Tinuvin PA 123; or (iii) a hybrid HALS/UV-absorber, composed by hydrophilic 2-(2-hydroxyphenyl)-benzotriazole as UV absorber and a basic HALS, with the commercial name of Tinuvin 5151. Figure 1 shows the chemical structure of the different UV-stabilizers used in this work, as well as the piperidine ring as a key structure in HALS.

The solvent-based lacquer-formulations were obtained by mixing two PUR commercial resins, a matting agent (silicone) and Tinuvin 770 DF or Tinuvin PA 123 as a UV-stabilizer, with final percentages of 96.6, 2.9 and 0.5% (m/m) for Tinuvin 770 DF and 96.9, 2.9 and 0.2% (m/m) for Tinuvin PA 123; while the water-based lacquer-formulations were obtained by adding 0.5% (m/m) of Tinuvin 5151 into a commercial waterborne PUR dispersion. Next, both formulations were spread with a 90 µm-thickness onto a polyethylene terephthalate (PET) inert surface, to produce lacquer-films. These films were further submitted to two heating cycles for solvent evaporation: 4 min at 50°C on a digital hotplate (Stuart SD300, R330000718) followed by 1 min at 140°C in a laboratory hot air oven type (Werner Mathis AG, LTF-ST 119988). All raw materials used to produce lacquer-films were purchased from commercial suppliers and are available at TMG Automotive. Before performing the antimicrobial and cytocompatibility assays, lacquer-films were submitted to a pre-treatment for 336 h (14 days) at 70°C in a drying and heating chamber (Model ED 400, Blinder^®^) to eliminate volatiles and solvent residues from the polymeric material. Lacquer-films of both solvent- and water-based formulations, without the incorporation of UV-stabilizers were also produced as control lacquer-films.

### 2.2 Evaluation of the antimicrobial activity of HALS-based lacquer-films

The antimicrobial assays were based on the protocol described in the International Organization for Standardization (ISO) 22,196(E):2011—Measurement of antibacterial activity on plastics and other non-porous surfaces (ISO22196:2011E, 2011). The assays were performed using two Gram-positive bacterial strains, *Staphylococcus aureus* ATCC 6538P and *Staphylococcus aureus* ATCC-BAA-2313 a methicillin-resistant bacteria; a Gram-negative bacterial strain *Escherichia coli* ATCC 8739; and a fungal strain *Candida albicans* SC5314/ATCC MYA-2876. Briefly, lacquer-films of 5 × 5 cm^2^ were inoculated with 0.4 mL of a bacterial or fungal suspensions (6 × 10^5^ CFU/mL). An inert cover-film (Ref. 132 225 of ©INTERSCIENCE) (4 × 4 cm^2^) was placed over the material surface to ensure the contact between inoculum and lacquer-film. Test samples were incubated for 24 h at 32°C for antifungal assays and 35°C for antibacterial assays, in an atmosphere with high relative humidity. Both lacquer- and cover-films were previously sterilized by UV-light for 30 min on each side. Immediately after inoculation, initial inoculum and samples of untreated material (control lacquer-films) were processed to validate the assay. After 24 h incubation, lacquer-films were washed with a wash solution composed by soybean casein, lecithin and polyoxyethylene sorbitan monooleate. Ten-fold serial dilutions of the microbial suspensions were performed in phosphate-buffered physiological saline solution (PBS). Each dilution, as well as the initial suspension, was plated on agar plate medium composed by yeast extract, tryptone and glucose for the antibacterial assays or by a yeast-extract, peptone and dextrose for the antifungal assays. The plates were incubated at 32°C or 35°C for 40–48 h. Following this incubation period, the number of CFU were counted. As described by Costa et al. (2023), the antimicrobial activity (R) was calculated by subtracting the average of common logarithm of the number of viable colonies recovered from untreated materials 24 h after inoculation and the average of common logarithm of the number of viable colonies recovered from treated materials after 24 h of inoculation. In addition, the microbial ratio (MR) was determined to quantify the percentage of the microbial burden present in each treated materials relative to the bacterial burden in untreated materials (control lacquer-films).

### 2.3 Evaluation of Tinuvin 770 DF mechanism of action: reagents and solutions

To evaluate the mechanism of action of Tinuvin 770 DF, different stock and work solutions were prepared. Specifically, Tinuvin 770 DF was prepared in isopropyl alcohol at 150 mg/mL. To test the contribution of the piperidine compound, a solution of 4-hydroxy-2,2,6,6-tetramethylpiperidine ≥98.0% (C_9_H_18_NO_2_, CAS no. 2403-88-5; TLC, Japan) in distilled water at 50 mg/mL was used. To test the contribution of RNS, a copper (II) chloride dihydrate ≥99.0% (CuCl_2_.2H_2_O, CAS no. 10125-13-0) solution prepared in distilled water at 800 μg/mL was used as a RNS-scavenger. A solution of 4-amino-5-methylamino-2′,7′-difluorofluorescein diacetate >95.0% (DAF-FM) (Sigma Aldrich) at 5 × 10^−6^ M in dimethyl sulfoxide was used as a nitric oxide-specific probe.

### 2.4 Evaluation of Tinuvin 770 DF mechanism of action: half-maximal inhibitory concentration (IC50) by broth microdilution assay

IC50 determination was performed using a microtiter plate colorimetric assay, based on resazurin reduction. Briefly, 100 µL of bacterial suspension (1 × 10^6^ CFU/mL) were incubated for 24 h in Muller-Hinton (MH) medium with increasing concentrations of Tinuvin 770 DF; 4-hydroxy-2,2,6,6-tetramethylpiperidine; or Tinuvin 770 DF with CuCl_2_.2H_2_O at a fixed concentration of 800 μg/mL. After this period, 20 µL of 0.02% resazurin dye was added to the wells. After 3–4 h of incubation, the optical density was determined spectrophotometrically at 575 and 610 nm (Infinite^®^ M200 NanoQuant Micro Plate Reader, Tecan Systems, Switzerland). IC50 was determined using the GraphPad Prism software (version 8.0.2) adjusting the experimental points to a nonlinear regression (curve fit). A positive control (MH medium with bacteria) and a negative control (only MH medium) were also included.

### 2.5 Evaluation of Tinuvin 770 DF mechanism of action: colony forming units (CFU) counting

Bacterial suspensions (1 × 10^5^ CFU/well) were incubated in a 96-well plate for 24 h, at 37°C, with increasing concentrations of Tinuvin 770 DF alone or in the presence of CuCl_2_.2H_2_O, to reach a final volume of 200 µL/well. At the end of the incubation period, 10-fold serial dilutions were performed in PBS-saline. Each dilution, as well the initial suspension, were plated on nutrient agar medium based on yeast extract, tryptone and glucose. The plates were incubated for 24 h at 37°C, after which CFU were counted. Results are expressed in percentage of bacterial burden from when compared with the positive control (bacteria culture alone).

### 2.6 Evaluation of Tinuvin 770 DF mechanism of action: nitric oxide quantification

Bacterial suspensions (1 × 10^5^ CFU/well) were incubated in a 96-well plate with increasing concentrations of Tinuvin 770 DF alone or in the presence of CuCl_2_.2H_2_O were incubated for 24 h at 37°C, to reach a final volume of 200 µL/well. After the incubation period, 1 µL of DAF-FM (5 × 10^−6^ M) was then added at each well and plates were incubated for an additional 30 min at 37°C in the dark. The intensity signal of DAF-FM was measured using the FACS LSRII flow cytometer (BD Biosciences^®^), with excitation laser of 488 nm. The resolution gates were adjusted according to each bacterial strain. At least 3,000 events were recorded for each experimental condition. Flow cytometric analysis was performed using the FlowJo 10.8.1 (Becton Dickinson & Company 2006-2021). The results are expressed as mean of fluorescence intensity (MIF) for at least 4 independent biological experiments.

### 2.7 Cytocompatibility of antibacterial lacquer-films

Cytocompatibility assays were performed using the above-described microtiter plate colorimetric assay and an adaption of the International Organization for Standardization (ISO) 10,993-5:2009 - Biological evaluation of medical devices, Part 5: Tests for *in vitro* cytotoxicity (ISO10993-5, 2009). Briefly, supernatant with lacquer extractable products was obtained by incubating both control and test lacquer-films with different concentrations of Tinuvin 770 DF (from 0.5% to 10% (m/m) in culture media (Dulbecco’s Modified Eagle’s Medium supplemented with 10% of FBS, and 1% of glutamine and HEPES buffer) at room temperature for 24 h under orbital shaking (100 rpm). In parallel, 100 µL of L-929 fibroblast cell line, at density of 1 × 10^5^ cells/mL (maximum passages of 10), were incubated in a 96-well plate, for 24 h, at 37°C, 5% CO2, and >90% humidity in an Heraeus HERAcell 150 Incubator (Thermo). After the incubation period, cell culture media was replaced with 100 µL of lacquer-extract medium and plates were incubated for an additional 24 h at 37°C. After 24 h, 20 µL of 0.02% resazurin dye was added at each well. To allow the resazurin reduction, 96-well plate were incubated for 3–4 h. The optical density was measured spectrophotometrically at 575 and 610 nm. A positive control (cells cultured in cell culture medium), a negative control (only cell culture medium) and a control of cytotoxicity (1% Triton X-100) were also included.

The results were expressed as percentage of cell viability from control lacquer-film, by the ratio of absorbance between at 575 and 610 nm for the different conditions, according to the following equation:

$$\%\;cell\;viability=\frac{Abs\;sample-Abs\;negative\;control}{Abs\;positive\;control-Abs\;negative\;control}\times 100$$

According to the ISO10993-5:2009 protocol, a cell viability below 70% is defined as positive cytotoxic activity (ISO10993-5, 2009). Following the protocol recommendations, a minimum of four test lacquer-films with increasing concentrations of Tinuvin 770 DF were used to assess cytocompatibility.

In addition, fluorescence microscopy techniques were used to assess the morphology of L-929 cells and confirm cell death. For this, supernatants containing lacquer extractable products were incubated for 24 h with the L-929 fibroblast cell line under identical conditions as described above. After incubation, Propidium Iodide (PI) at 50 μg/mL was added to each well. Subsequently, the 96-well plate was incubated for 15 min at room temperature in the dark. Cells were then observed with the EVOS FLoid Invitrogen microscopy using a red channel (λ_exc_ = 515/586 nm and λ_emi_ 646/668 nm). The different experimental conditions were photographed (bright-field and with red light-filter) using an integrated Sony 1.3 MP 1/3” ICX445 EXview HAD CCD camera. Positive and negative controls, as well as a control of cytotoxicity, were included for comparison.

### 2.8 Statistical analysis

Data is reported as mean ± standard deviation. Statistical analysis was done using GraphPad Prism (version 8.0.2) by testing the normality (Shapiro-Wilk test) and the significance values. Unpaired t-test was used for antimicrobial lacquer-films analysis. Data was considered statistically significant for *p* < 0.05.

## 3 Results

UV-stabilizers have been used for several years with the main purpose of restricting UV penetration into the material and ultimately improve durability and/or delay degradation by photo-oxidation. In addition to these features, some UV-stabilizers, such as HALS, have been associated to microbial growth restriction, although the exact mechanism(s) of action have not been fully explored. To further investigate the antimicrobial effect of specific UV-stabilizers, Tinuvin 770 DF, Tinuvin PA 123 and Tinuvin 5151 were selected and incorporated into a PUR commercial resin to produce lacquer-films.

### 3.1 Antimicrobial performance of produced lacquer-films

Our first step was to evaluate the antimicrobial activity of different HALS, specifically Tinuvin 770 DF, Tinuvin PA 123 and, Tinuvin 5151. For that purpose, these UV-stabilizers were incorporated into different commercial PUR resins and formulated to produce solvent- or water-based lacquer-films. The lacquer-films were produced using the solvent casting method by spreading the polymer solution onto an inert PET surface. The concentration of the additives follows typical commercial concentrations and are aligned with the literature, which describes concentrations of UV-stabilizers to be around 0.05%–2% (m/m) (Chin, 2007). The antimicrobial properties of lacquer-films were tested against two Gram-positive bacterial strains (*S. aureus* methicillin and non-methicillin resistant strains), a Gram-negative bacterial strain (*E. coli*) and a fungal strain (*C. albicans*), using the international protocol, ISO 22196(E):2011.

**FIGURE 2:**
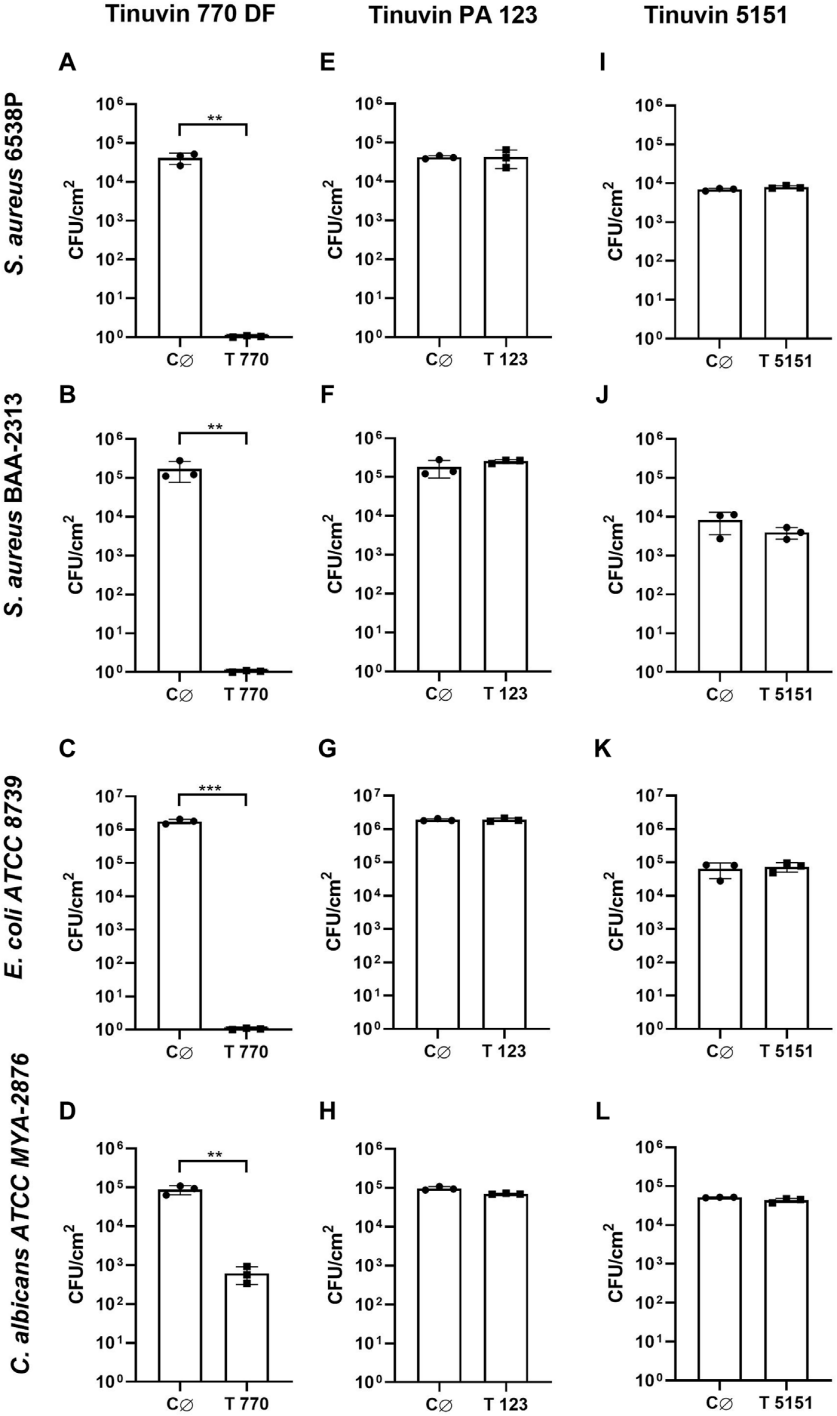
Antimicrobial activity of lacquer-films. **(A–D)** Antimicrobial analysis of Tinuvin 770 DF solvent-based lacquer-films against **(A)** *S. aureus* 6538P, **(B)** *S. aureus* BAA-2313 **(C)** *E. coli* ATCC 8739 and **(D)** *C. albicans* ATCC MYA-2876. **(E, H)** Antimicrobial analysis of Tinuvin PA 123 solvent-based lacquer-films against **(E)** *S. aureus* 6538P, **(F)** *S. aureus* BAA-2313 **(G)** *E. coli* ATCC 8739 and **(H)** *C. albicans* ATCC MYA-2876. **(I–L)** Antimicrobial analysis of Tinuvin 5151 water-based lacquer-films against **(I)** S. *aureus 6538P*, **(J)** *S. aureus* BAA-2313 **(K)** *E. coli* ATCC 8739 and **(L)** *C. albicans* ATCC MYA-2876. C∅ represents the control lacquer-films and T 770, T 123 and T 5151 represents the antimicrobial lacquer-films with incorporation of UV-stabilizers. Error bars represent the standard deviation (n = 3). Unpaired t-test: **p < 0.01 and ***p < 0.001.

For lacquer-films incorporated with Tinuvin 770 DF no viable bacteria were recovered from the test surface, corresponding to an R value > 4 for Gram-positive strains and an R value > 6 for the Gram-negative bacteria (Figure 2A–C). These results show a reduction of over 99.99% for the tested bacterial strains (Table 1). For *C. albicans*, the Tinuvin 770 DF lacquer-films showed an R value of 2.19 representing a fungal reduction of 99.31% (Figure 2D; Table 1). Regarding the lacquer-formulations incorporated with Tinuvin PA 123 or Tinuvin 5151, the number of viable bacteria and fungi recovered from the control and test lacquer-films were similar, indicating no antimicrobial activity, regardless the microorganism (Figure 2E–L; Table 1).

**TABLE 1:** Antimicrobial activity of produced lacquer-films formulated with different UV-stabilizers.

		*S. aureus* 6538P		*S. aureus* BAA-2313		*E. coli 8739*		*C. albicans ATCC MYA-2876*	
		R value^a^	MR (%)**	R value^a^	MR (%)**	R value^a^	MR (%)**	R value^a^	MR (%)**
Solvent-based lacquer-formulation	Tinuvin 770 DF	4.60	>99.99	5.20	>99.99	6.25	>99.99	2.19	99.31
	Tinuvin PA 123	0.03	4.52	−0.21	−32.25	−0.03	−5.81	0.11	21.51
Water-based lacquer-formulation	Tinuvin 5151	−0.06	−14.59	0.26	52.10	−0.09	−15.26	0.08	15.38

^a^ Antibacterial and antifungal activity (R) is calculated according to ISO, 22196:2011 guidelines, by difference between the average of the common logarithm of the number of viable bacteria recovered 24 h of incubation for control lacquer-films and the average of the common logarithm of the number of viable bacteria recovered 24 h of incubation for tested lacquer-films (lacquer-films with Tinuvim 770 DF, or Tinuvim 5151. ** Microbial Ratio (MR) is the percentage of viable bacteria recovered from treated samples compared to untreated samples after 24 h of incubation. Negative values of Rvalue or MR, indicate an increase of bacterial load compared to control lacquer-films.

### 3.2 IC50 analysis of Tinuvin 770 DF

Based on the previous findings that lacquer-films incorporated with Tinuvin 770 DF show a strong antimicrobial spectrum of action, our next step was to determine its possible mechanism of action. In the literature, the impact of HALS on microbial growth inhibition is limited, despite some studies suggesting that the antimicrobial activity could be related to either the action of piperidines by protonation to a quaternary ammonium or the oxidation of nitroxyl free radicals (Paine et al., 2016). The piperidine ring is described as an important component of HALS, being essential not only for its function as a UV-stabilizer, but also to ensure its compatibility with other additives in the polymeric matrix through the functionalization of the aromatic ring (Liauw et al., 2004), as depicted by its chemical structure in Figure 1D. Another important feature in HALS structure is their secondary amine, which can form a nitroxyl radical acting as an intermediate step in the capture of alkyl radicals (Hintersteiner et al., 2014).

**FIGURE 3:**
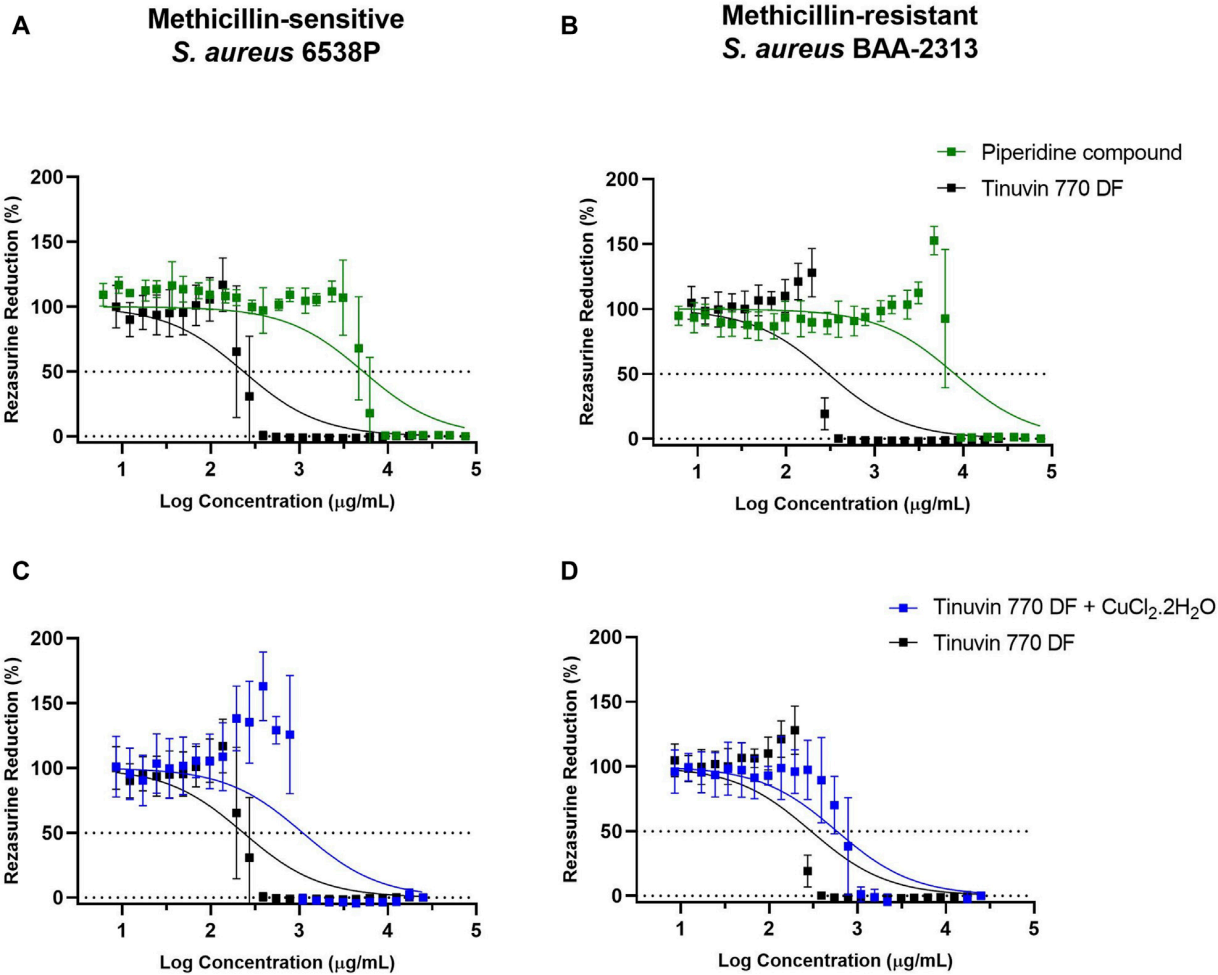
IC50 analysis of selected compounds: **(A, B)** IC50 analysis of Tinuvin 770 DF (dark line) and 4-Hydroxy-2,2,6,6-tetramethylpiperidine (green line) against **(A)** *S. aureus* 6538P and **(B)** *S. aureus* BAA-2313. **(C, D)** IC50 analysis of Tinuvin 770 DF (dark line) and Tinuvin 770 DF in the presence of RNS/ROS scavenger (blue line), against **(C)** *S. aureus* 6538P and **(D)** *S. aureus* BAA-2313 **(D)**. Horizontal lines represent the resazurin reduction at 50%. Error bars represent the standard deviation for at least 3 independent experiments with triplicates.

We started by determining the IC50 of the Tinuvin 770 DF. For this analysis, we only focused on methicillin-sensitive and methicillin-resistant *S. aureus* strains (MSSA and MRSA, respectively), due to their high predominance on inanimate surfaces and their importance in public health as one of the most relevant human pathogenic bacteria (Wißmann et al., 2021). IC50 analysis was performed based on resazurin reduction (blue dye) to resorufin (pink color) by viable cells, which measures the metabolic activity of viable cells (Schmitt et al., 2013; McGaw et al., 2014). Data demonstrated that Tinuvin 770 DF exhibited an IC50 value of 233.8 and 298.0 μg/mL, against MSSA and MRSA, respectively (Figure 3).

Firstly, to explore the possibility that piperidine rings are responsible for the mechanism of action of HALS we tested the antibacterial activity of 4-hydroxy-2,2,6,6-tetramethylpiperidine. The IC50 value for the piperidine compound against MSSA was significantly higher than Tinuvin 770 DF (5,268 μg/mL vs. 233.8 μg/mL), representing a more than 22-fold increase (Figure 3A). The same pattern was observed for MRSA (Figure 3B), with the piperidine compound’s IC50 being more than 26-fold higher (8,013 μg/mL) than the IC50 obtained with Tinuvin 770 DF (298.0 μg/mL). Overall, these results suggest that the antimicrobial action of Tinuvin 770 DF was not associated to the action of piperidines, at least as an isolate compound.

Next, we hypothesized whether the generation of reactive nitrogen species (RNS) could underly Tinuvin 770 DF antimicrobial properties. For that we incubated MSSA or MRSA strains suspensions with Tinuvin 770 DF in the presence or absence of the RNS scavenger, CuCl_2_.2H_2_O (Klotz et al., 2003; Goel et al., 2016; Majumdar and Kar, 2018). Copper can act as a scavenger for RNS and other reactive species (H_2_O_2_ and O^2−^) because of their strong quantum confinement of electrons (Chiarantino et al., 2003; Liu et al., 2020). The presence of unpaired electrons in copper’s atomic structure promotes the electron transfer between the metal and the ligand (NO, H_2_O_2_ and/or O_2_), ultimately inactivating them (Chiarantino et al., 2003; Liu et al., 2020). Importantly, when the RNS scavenger was added together with Tinuvin 770 DF, there was an approximately 5-fold increase in the IC50 value (1,093 μg/mL) against MSSA (Figure 3C). Similar results were obtained with MRSA, in which the IC50 of Tinuvin 770 DF alone increased from 289 μg/mL to 584 μg/mL in the presence of the RNS scavenger (Figure 3D). It is noteworthy that the concentration of CuCl_2_.2H_2_O used in this study (800 μg/mL) did not have a significant effect on bacterial viability (our previous studies show a MIC value of 970 μg/mL for CuCl_2_.2H_2_O against both bacterial strains–data not shown). Based on the reduced antimicrobial activity of Tinuvin 770 DF when in the presence of CuCl_2_.2H_2_O (Figure 2E, F), these results suggest a possible role of RNS in the inhibition of bacterial growth.

### 3.3 Impact of antibacterial properties of Tinuvin 770 DF in the presence of RNS scavenger

To further confirm the role of RNS in the antibacterial performance of Tinuvin 770 DF, we evaluated the production of NO in parallel with the percentage of bacterial death induced by Tinuvin 770 DF. In this work, NO species were measured by flow cytometry with the NO-sensitive probe DAF-FM that crosses the cellular membranes by passive diffusion and is converted to a fluorescent triazole derivative in the presence of RNS, in particular NO species (Cottet-Rousselle et al., 2011; Namin et al., 2014; Griendling et al., 2016).

**FIGURE 4:**
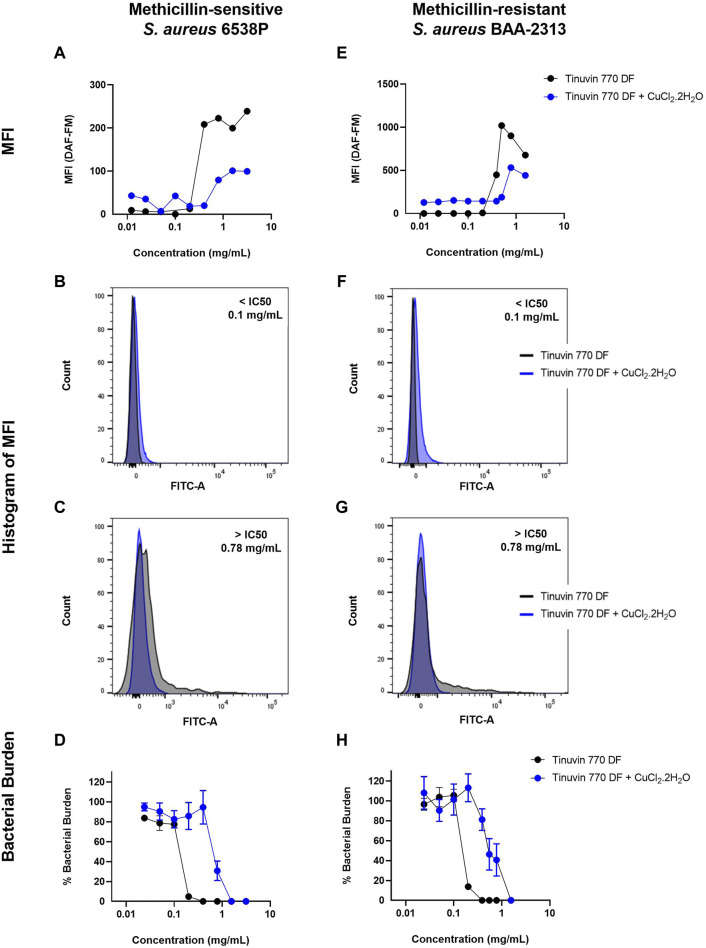
DAF-FM Nitric oxide quantification and antibacterial performance of Tinuvin 770 DF. MFI values of Tinuvin 770 DF (dark line) and Tinuvin 770 DF in the presence of RNS/ROS scavenger (blue line) against **(A)** *S. aureus* 6538P and **(E)** *S. aureus* BAA-2313 (MRSA). Histogram of MFI for Tinuvin 770 DF alone and in the presence of RNS/ROS scavenger against *S. aureus* 6538P at **(B)** 0.1 mg/mL and **(C)** 0.78 mg/mL. Histogram of MFI for Tinuvin 770 DF alone and in the presence of RNS/ROS scavenger against *S. aureus* BAA-2313 (MRSA) at **(F)** 0.1 mL/mL and **(G)** 0.78 mg/mL; CFU analysis of Tinuvin 770 DF and Tinuvin 770 DF in the presence of RNS/ROS scavenger against **(D)** *S. aureus* 6538P and **(H)** *S. aureus* BAA-2313 (MRSA). Error bars represent standard error of the mean for at least 4 independent biological replicates.

Data showed that incubation of MSSA with low concentrations of Tinuvin 770 DF [10–200 μg/mL] resulted in basal levels of NO (MFI)∼10), while at higher concentrations of Tinuvin 770 DF [400–3,130 μg/mL] a 15-fold increase in DAF-FM MFI was observed (Figure 4A). Indeed, at a concentration of Tinuvin 770 DF above the IC50 value, there was a clear shift in the histogram profile, when compared to the histograms presented by bacteria treated with a concentration below the IC50 value (Figure 4B, C). Importantly, these high levels of NO were associated with a complete bacterial elimination of MSSA when incubated with Tinuvin 770 DF at concentrations above 400 μg/mL (Figure 4D). On the other hand, lower levels of NO corelated with viable bacteria, ranging from 4.8% to 83.7%, when Tinuvin 770 DF was used at concentrations below the IC50 (Figure 4D). Regarding MRSA, the results were similar to those observed for MSSA (Figure 4E–H). At concentrations lower than the IC50 (below 200 μg/mL), residual DAF-FM MFI (MIF∼10) was detected while at higher concentrations of Tinuvin 770 DF [400–1,560 μg/mL] there was an increase in NO accumulation, reaching a maximum MFI value of 1,019 (Figure 4E–G). Importantly, this increased accumulation of RNS correlated with a decrease in the number of viable bacteria, regardless the strain (Figure 4H).

Importantly, the addition of the RNS scavenger, CuCl_2_.2H_2_O, to either the MSSA or MRSA suspensions treated with increasing concentrations of Tinuvin 770 DF resulted in a reduction of the DAF-FM MFI, indicating a decrease in the accumulation of NO when compared to Tinuvin 770 DF alone (Figure 4A, E). Specifically, concentrations of Tinuvin 770 DF above the IC50 value, when incubated with MSSA or MRSA, resulted in an MFI∼222 or 900, respectively. When in the presence of CuCl_2_.2H_2_O, there was a clear shift in this histogram profile, resulting in a decreased MFI (79 and 531, for MSSA or MRSA, respectively) (Figures 4C,G) and an increased bactericidal concentration (Figure 4D, H). As expected, the addition of CuCl_2_.2H_2_O did not affect the levels of NO when bacteria were incubated with low levels of Tinuvin 770 DF (below IC50) (Figure 4B, F).

Together, the above-described results suggest that there was an increased production of RNS in the experimental conditions where Tinuvin 770 DF was present, most likely originating from its autoxidation process. Importantly, the levels of detected RNS inversely correlated with the percentage of viable bacteria.

### 3.4 Cytocompatibility of antimicrobial lacquer-films

**FIGURE 5:**
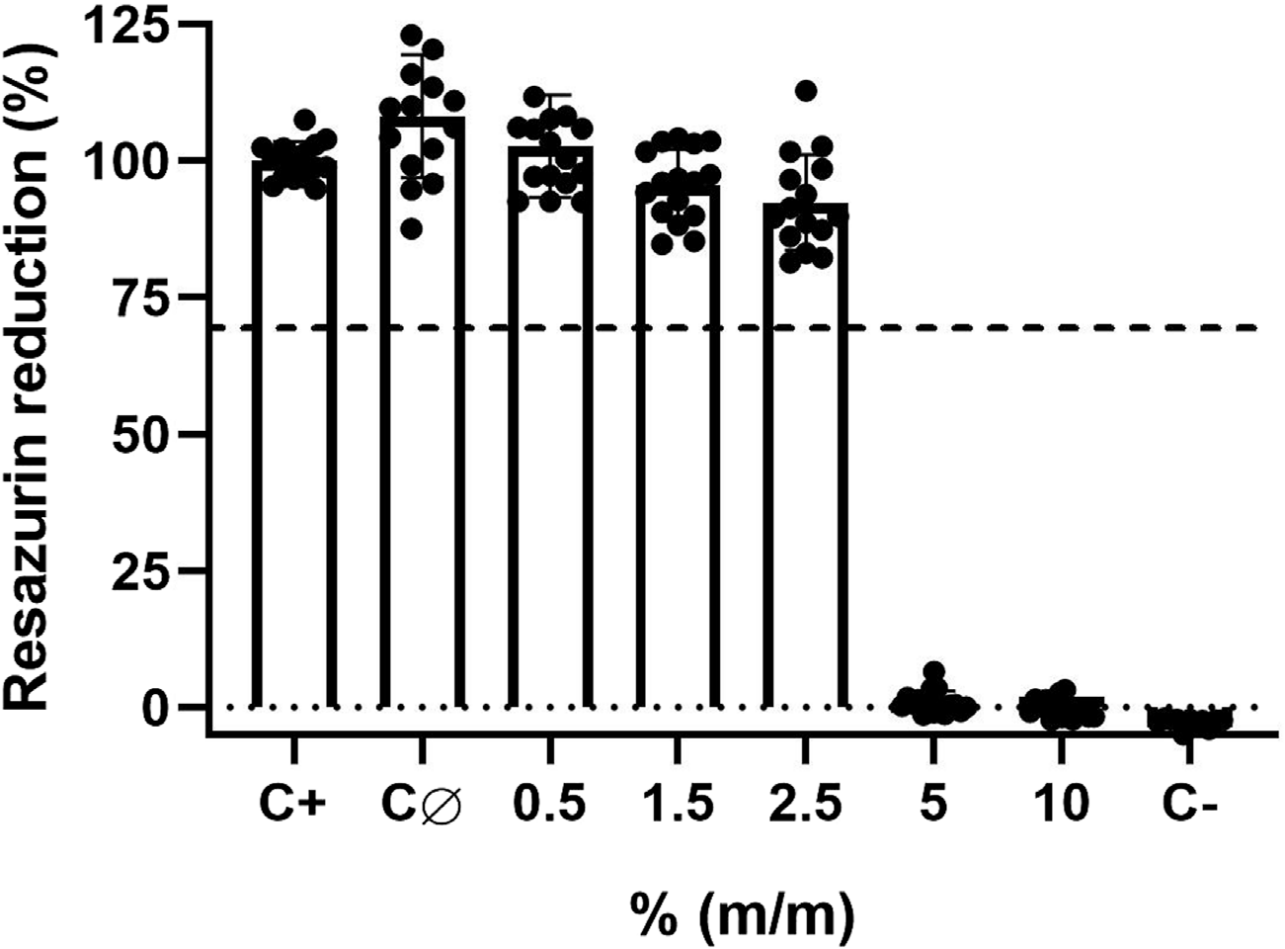
Cytocompatibility assays of solvent-based lacquer-films with different concentrations of Tinuvin 770 DF against L-929 mouse fibroblast cells: C+ represents the positive control (only cells in medium); CØ represents the control lacquer-films without Tinuvin 770 DF (control lacquer-film) and C- represents the cells exposed to Triton X-100 (negative control). Dashed line represents 70% of resazurin reduction. Error bars represent the standard deviation of 4 independent biological assays with quadruplicates in each assay.

The cytocompatibility was determined by indirect exposure of L-929 mouse fibroblast to lacquer-extract medium, using an adaptation of ISO 10993-5:2009 protocol (ISO10993-5, 2009). As expected, results demonstrated that control lacquer-films without Tinuvin 770 DF had a high cytocompatibility with more than 100% of cell viability (Figure 5). Furthermore, lacquer-films with 0.5%, 1.5%, and 2.5% (m/m) of incorporated Tinuvin 770 DF also showed cytocompatibility, with percentages of cell viability of 102.7% ± 9.0%, 95.5% ± 6.3% and 92.4% ± 8.4%, respectively. Nevertheless, incorporation of more than 5% (m/m) of Tinuvin 770 DF in the lacquer-films resulted in a significant cytotoxic effect with cell viability near 0%. It should be noted that for antimicrobial studies, we use a concentration of Tinuvin 770 DF of 0.5% (m/m) (within the commercial concentration range), for which this UV-stabilizer has an antimicrobial effect and also high cytocompatibility.

**FIGURE 6:**
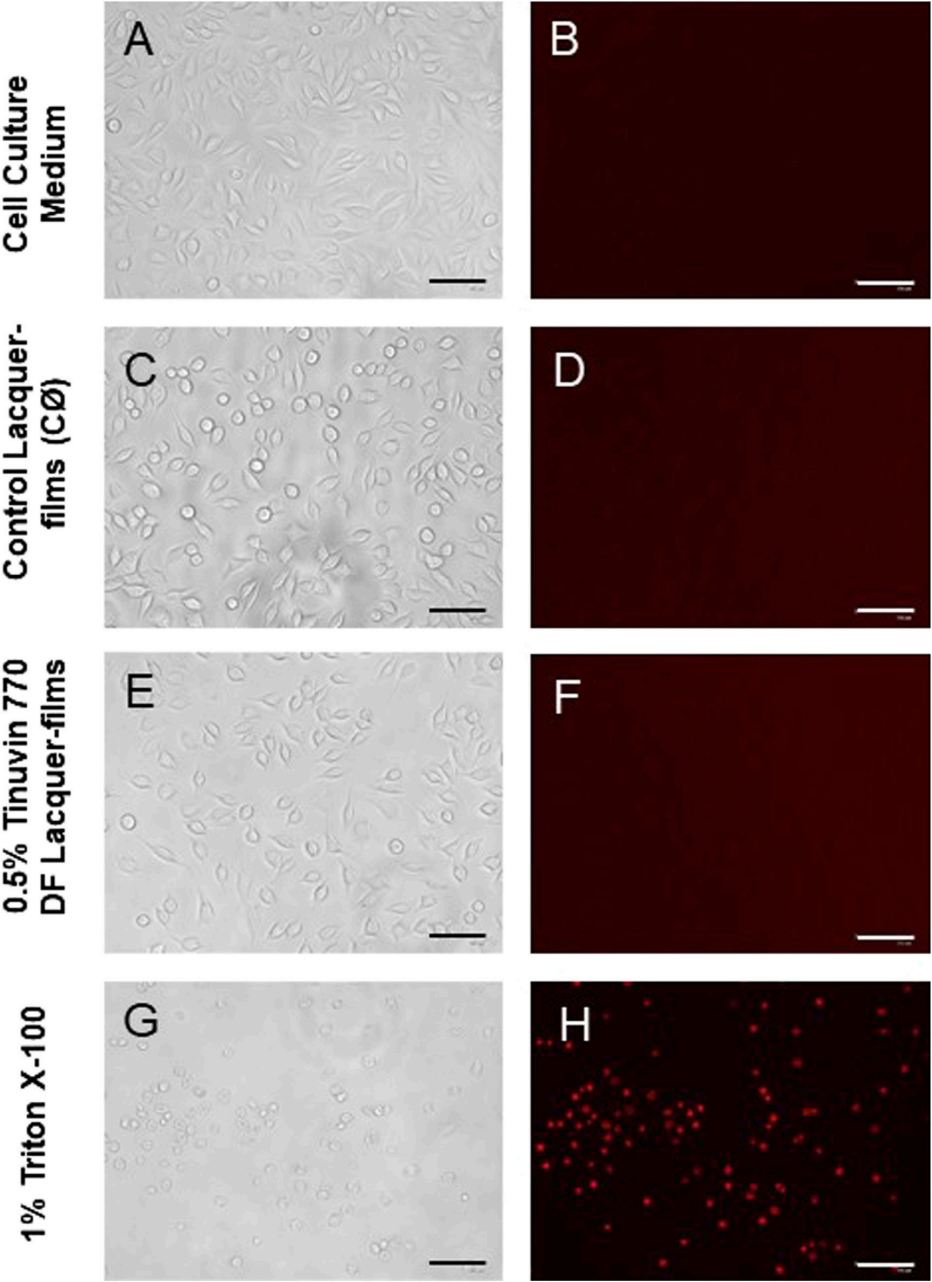
Microscopy images of L-929 mouse fibroblast cell line: **(A, B)** L-929 cells incubated with DMEM (positive control, C+); **(C, D)** L-929 cells exposed to extractable products from control lacquer-films (CØ); **(E, F)** L-929 cells exposed to extractable products from lacquer-films incorporated with 0.5% (m/m) of Tinuvin 770 DF; and **(G, H)** L-929 cells exposed to Triton X-100 (negative control, C-). The columns represent the different acquisitions methods: **(A, C, E, and G)** bright-field; and **(B–H)** PI staining. Scale bar = 125 µm.

To further confirm these results, microscopy images were acquired to assess cell morphology and viability after exposure to extractable medium from lacquer-films (Figure 6). In Figure 6A, it is possible to observe a cell-adherent monolayer with a morphology typically associated to fibroblasts. A similar cell morphology was observed for cells exposed to extracted medium from control lacquer-films (Figure 6C) or lacquer-films incorporated with 0.5% (m/m) of Tinuvin 770 DF (Figure 6E). On other hand, cells exposed to 1% of Triton X (Figure 6G) were mostly detached and presented a round shape, morphological features commonly associated to cell death. This observation was confirmed with PI staining. PI is a fluorescent dye capable of biding to DNA of membrane-damaged cells and is therefore commonly used as an indicator of cell death (Crowley et al., 2016; Gutiérrez et al., 2017). The lack of fluorescence signal for cells exposed to extractable medium from 0.5% (m/m) Tinuvin 770 DF lacquer-films, when compared to the intense fluorescent signal in cells exposed to 1% of Triton (Figure 6H), further confirms the cytocompatibility of Tinuvin 770 DF lacquer-films (Figure 6F).

## 4 Discussion

Polymer-based materials are versatile materials used in numerous industries, including packaging, automotive, construction, electronics, and healthcare. Their main properties are determined by the incorporation of polymer additives during their processing. Specifically, these additives can control/improve processing, promote specific properties of interest, induce aesthetic effects, and prevent/avoid polymer degradation (Marturano et al., 2019). Degradation can be defined as all processes which lead to a decline of polymer properties, and can be caused by heat, light, oxidation, hydrolysis and, exposure to chemicals (Marturano et al., 2019) as well as by the action of microorganisms (Jasso-Gastinel et al., 2017). Light, and in particular UV-radiation, can promote the loss of structural integrity of polymeric chains and induce discoloration of materials, such as yellowing of polymers, loss of gloss and loss of mechanical properties (e.g., cracking) (Yousif and Haddad, 2013). To avoid light-associated polymer degradation, UV-stabilizing additives are commonly used acting by reducing/limiting the amount of radiation that reaches the polymer or by blocking the chemical reactions induced by absorbed radiation. Specifically, UV-stabilization methods include: (i) blockade or absorption of UV radiation before it can reach the chromophore(s); (ii) deactivation or quenching of the excited species originated by the UV radiation and its conversion into stable or non-reactive forms; or (iii) scavenging of free radicals originated by UV radiation and its conversion into stable or non-reactive forms (Chin, 2007). Regarding the impact of microorganisms, biodegradation occurs by reduction of polymer chains into low molecular weight constituents, followed by surface and bulk erosion of the polymeric materials (Brdlík et al., 2022). In particular, the biodegradation of PUR occurs by cleavage of urethane bonds (Mohanan et al., 2020). Moreover, PUR can act as a carbon and urease substrate for fungi and bacteria, increasing its susceptibility to microbial colonization and ultimately biodegradation (Pathak and Navneet, 2017). Considering this, the development of polymeric materials with additives with dual function (UV-stabilizer and biocide agent) has been gaining interest as an attractive solution to avoid both photo- and biodegradation of polymers.

Interestingly, UV-stabilizers such as HALS, have been reported to have an impact on microbial colonization. Based on this information, in the present work, three commercial UV-stabilizers based on HALS were incorporated into PUR-formulations to produce lacquer-films: Tinuvin 770 DF, Tinuvin PA 123, and Tinuvin 5151, a hybrid HALS/UV-absorber. The lacquer-films incorporated with Tinuvin 770 DF presented a significant reduction in the microbial load for both Gram-positive and Gram-negative bacteria, as well as for fungal species, showing a broad spectrum of action. On the other hand, the lacquer-films incorporated with Tinuvin PA 123 and Tinuvin 5151 did not show any antimicrobial activity. Considering that the differences in the chemical structures among the tested HALS are mostly related to the piperidine ring (Figure 1A–C), these structural differences may be related to antimicrobial activity. Specifically, the amine group in the piperidine ring of the Tinuvin 770 DF structure is not linked to any additional group, while in Tinvin PA 123, the piperidine ring is linked to an additional alkyl chain. Tinuvin 5151, on the other hand, contains an extra CH_3_ group linked to the amine group in the piperidine ring. Given that the amine group is one of the most critical functional groups in HALS, variations in the “degree of freedom” may account for differences its antibacterial activity. Indeed, Tinuvin 770 DF is the only HALS tested that exhibits antimicrobial activity, possibly due to its increased freedom in the amine group, which may enhance its propensity for oxidation. In the literature there are some reports related to the antimicrobial activity of HALS-based structures but, generally they are combined with other chemical groups, mainly chloride reactive groups. Indeed, Abualnaja et al. (2021) reported the development of HALS-based compounds containing chromophores with dual function: photo-stability and antimicrobial activity, which presented a moderate biological activity with antimicrobial reduction of 9% for *C. albicans* and 17%–24% against *S. aureus* and *E. coli*, respectively (Abualnaja et al., 2021). In another work, Li et al. (2014) reported the development of a chlorinated-HALS, based on 2,2,6,6-tetramethyl piperidinol (followed by a post-chlorination process) incorporated in cotton fabric. The chlorinated-HALS had strong antibacterial properties against *S. aureus* (100% bacterial reduction), but moderate antibacterial activity against *E. coli* (83.25% bacterial reduction), after only 10 min of contact. For the same conditions, unchlorinated-HALS showed a moderate bacterial reduction for *S. aureus* (87.3%) and null bioactivity for *E. coli* (6.2% reduction) (Li et al., 2014). In a similar approach, Paine et al. (2016) developed a polyester based material with 2% (m/m) of Tinuvin 770 DF or a modified Tinuvin 770 DF with a post-chlorination process and tested their antibacterial activity against *P. aeruginosa*. They found a decrease in bacterial protein quantification with N-chlorinated HALS, in comparison with Tinuvin 770 DF alone. However, they did not observe antifungal properties against *Cladosporium* spp. (Paine et al., 2016). Furthermore, Chen and Sun (2005) reported the production of a HALS based on a bis(N-chloro-2,2,6,6-tetramethyl-4-piperidinyl) sebacate (Cl-BTMP) in a polypropylene substrate. Authors showed that films with 0.1% (m/m) of Cl-BTMP effectively killed Gram-positive and Gram-negative bacteria after 15 min of contact. However, both BTMP (concentration between 0.1% and 4% (m/m)) and substrate alone did not have antibacterial effect (Chen and Sun, 2005).

**FIGURE 7:**
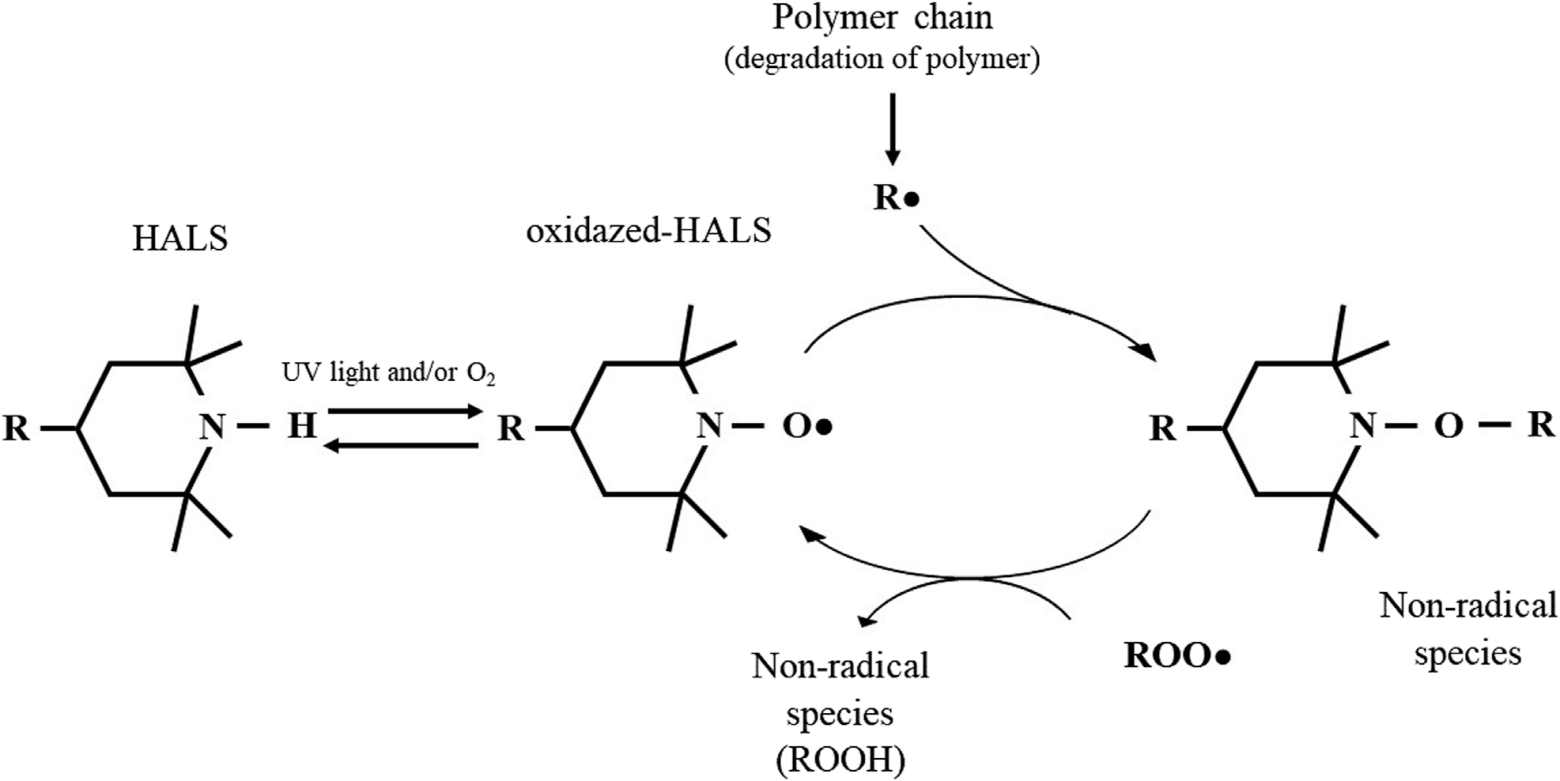
Mechanism of action of HALS based on the Denisov Cycle: HALS act by eliminating free radicals resulting from polymer degradation. The nitroxide reactive species -formed by oxidation of HALS through light or in the presence of oxygen: RNO• radicals - react with other radical species to form intermediate non-radical species. In turn, these intermediate products are capable of reacting with other radical species to regenerate nitroxide. Adapted from Yousif and Hasan, 2013; Hodgson, and Coote, 2010; Babaghayou, et al., 2021.

Despite the described biological activity of HALS-based compounds, the antimicrobial mechanism of HALS is not well elucidated, but has been suggested to be related to their primary function as a UV-stabilizer, avoiding photo-degradation of polymers. Photo-degradation begins with the cleavage of the homolytic polymeric bonds (R-R) (Hodgson and Coote, 2010), a process that can occur either by (i) photochemical cleavage of aldehydes and ketones (known as Norrish type I reaction) (Chen, 2017; Albini, 2021) or (ii) hydrogen abstraction by an atmospheric radical, both resulting in the formation of a polymeric radical (R•). In turn, this R• can react with the atmospheric oxygen to form a peroxyl radical (ROO•). Finally, this ROO• can abstract a hydrogen atom from the polymer backbone inducing the hydroperoxide (ROOH) formation and more R•, promoting the degradation of the polymer (Hodgson and Coote, 2010). As described above, HALS are long term stabilizers that act by trapping the free radicals produced during the photo-oxidation process, protecting the polymer against photo-oxidative damages. This protective effect arises from the scavenger ability of HALS to form nitroxide radicals, in a process known as the Denisov cycle (Hodgson and Coote, 2010; Ambrogi, et al., 2017; Babaghayou, et al., 2021). The Denisov cycle begins with the oxidation of HALS into nitroxide radicals (RNO•) by peroxides or hydroperoxides. The RNO• can react with polymeric alkyl radicals (R•) forming non-radical products (NOR). After this, new RNO• can be generated from hydroxylamine ethers with alkylperoxy and acylperoxy radicals, continuing the cycle (Hodgson and Coote, 2010). In other words, the polymer is protected by HALS through the inhibition of free radical activity during thermal and/or oxidative reactions (Babaghayou et al., 2021). A simplified HALS based Denisov cycle is presented in Figure 7. Specifically, in the case of Tinuvin 770 DF, the amine 2,2,6,6-tetramethylpiperidine is oxidized into the 2,2,6,6-tetramethylpiperidin-1-loxyl (TEMPO), forming a nitroxide that reacts with the R• generated during photodegradation. This results in an alkoxylamine that in turn reacts with a peroxyl radical to re-establish the nitroxide species, as well as nonradical products (Hodgson and Coote, 2010). Given the formation of NO-radicals during the Denisov cycle, and the role of these radicals with a broad spectrum of antimicrobial activity against Gram-positive and Gram-negative bacteria (Hall, Jackson et al., 2020), fungi, and viruses (Wiegand et al., 2021), we hypothesized that NO-radicals may be involved in the antimicrobial mechanism of Tinuvin 770 DF. Tinuvin PA 123 and Tinuvin 5151 incorporated in lacquer-films were shown not to have antimicrobial activity most likely due the decreased reactivity of their amine group to autoxidation. Indeed, in the case of Tinuvin PA 123 there are an oxygen atom and an alkyl chain linked to the N, whereas in the case of Tinuvin 5151 there is a methyl group linked to the N (Figure 1A–C).

NO is a small molecule with lipophilic and hydrophilic behaviour, being able to cross biological membranes (Schairer et al., 2012; Chen et al., 2022). It plays a crucial role in many biological processes, acting as a signalling molecule for cellular growth and metabolic activity; but it can also act as an effector molecule that actively kills invading microorganisms and tumour cells during the inflammatory process (Rong et al., 2019). At high concentrations, NO has an antimicrobial effect by oxidizing lipids, proteins, and nucleic acids, altering their structural and functional properties and promoting oxidative and nitrosative stress (Chen and Sun, 2005; Schairer et al., 2012). Due to their antimicrobial potential, NO-releasing polymers have been developed for many applications (Rong et al., 2019) in a wide range of substrates, including PET and silicone elastomer (SE) (Fleming et al., 2017); poly (ether-block-amide) (PEBA) and nylon (Wang et al., 2018); PUR-based materials with selenium (Qu et al., 2019); and polyvinyl chloride (Hopkins and Frost, 2018).

In this work, we used DAF-FM, as a NO-specific probe, to analyse the cellular NO-content after exposure of bacterial suspension to a range of concentrations of Tinuvin 770 DF. We focused on its antimicrobial activity against MSSA and MRSA due to their high incidence as one of the most important human pathogens and their high persistence on inanimate surfaces (Wißmann et al., 2021). In fact, *Staphylococcus* species are responsible for the colonization of a large range of surfaces (including in healthcare facilities (Menezes et al., 2022) fitness gym facilities (Dalman et al., 2019), automobile interiors (Stephenson et al., 2014)) and cloths, plastics, steel, glass or flooring materials (Wißmann et al., 2021). Our results show that there is an association between NO-content and bacterial viability. High levels of NO are associated with higher Tinuvin 770 DF concentrations and no viable bacteria. On the other hand, lower Tinuvin 770 DF concentrations exhibit a relatively low NO-content and high bacterial viability. This suggests that for low Tinuvin 770 DF concentrations, the NO produced does not have a toxic effect. Further reinforcing the importance of NO in the antimicrobial activity of Tinuvin 770 DF, is the fact that, in the presence of a NO-scavenger, a higher concentration of Tinuvin 770 DF was needed to decrease the bacterial load.

A critical issue that should be taken into consideration during the development of polymeric materials is their cytotoxic effect, specifically applications with the potential of contacting the skin. The cytotoxic activity of Tinuvin 770 DF lacquer-films was tested by exposing the mouse fibroblast L-929 cell line to lacquer-extract medium for 24 h at 37°C. The results show that the produced PUR-based lacquer-films exhibited no cytotoxic potential, up until 5% (m/m) of Tinuvin 770 DF. This concentration is 2.5-fold higher than the top concentration limit of HALS generally used in commercial formulations (Chin, 2007). Accordingly. L-929 cells exposed to extractable medium from lacquer-films incorporated with 0.5% (m/m) of Tinuvin 770 DF displayed normal fibroblast like-morphology with a spindle shape (Qian et al., 2018) and stained negatively with PI, confirming non-cytotoxic potential of these films. These results are aligned with the literature, which describes both PUR and HALS s being biocompatible. Specifically, PUR-based materials are described as biocompatible materials, being used in pharmaceutical and biomedical applications such as drug delivery applications, cardiovascular devices, scaffolds for tissue engineering and bone regeneration, and wound dressing (Wang and Wang, 2012; Halima et al., 2018; Uscátegui et al., 2019; Safina et al., 2021). Regarding biocompatibility of Tinuvin 770 DF the literature is limited. Nevertheless, some HALS have been described as being biocompatible, as is the case for Tinuvin PA 123 for S-G epithelial cell line and human gingival fibroblasts (Tipton et al., 2008).

The selection of additives is a crucial step in the polymer industry, since additives can modify specific functional properties and ultimately impact the final function of the material (Apicella et al., 2019). For the selected UV-stabilizers, we tested physical and chemical parameters to evaluate the impact of incorporation of Tinuvin 770 DF in solvent-based PUR lacquer-formulations (Supplementary Material). Based on the results regarding macroscopic and microscopic imaging, the timing of runoff (to analyse the impact on viscosity of liquid solution), FTIR and Raman spectroscopy analysis (to verify the chemical stability), mechanical testing and gloss analysis, we concluded that, as expected, the incorporation of Tinuvin 770 DF did not impact the properties of the prepared PUR-based lacquer-formulations and films (Supplementary Material).

## 5 Conclusion

In conclusion, this work highlights the potential of incorporating additives with dual function in polymeric materials. In particular, the Tinuvin 770 DF can act as a UV-stabilizer avoiding the photo-degradation of polymeric materials, as well as a biocide agent by inhibiting bacterial growth. Moreover, we highlight a potential antimicrobial mechanism of HALS, based on formation of NO species, during the photo-oxidation of Tinuvin 770 DF. Our results contribute to better understand the antimicrobial mechanism of HALS and possibly to expand the commercial application of Tinuvin 770 DF not only as a UV-stabilizer but also as a biocidal agent. In addition, understanding the exact mechanism of Tinuvin 770 DF can contribute to expand this knowledge to other UV-stabilizers with the same chemical reactive group or similar chemical properties.

## Data Availability

The raw data supporting the conclusion of this article will be made available by the authors, without undue reservation.
