# Supplementary material for: Antimicrobial properties of hindered amine light stabilizers in polymer coating materials and their mechanism of action
Source: Front Bioeng Biotechnol. 2024 Jun 24;12:1390513. doi: 10.3389/fbioe.2024.1390513 (PMC11229053; doi:10.3389/fbioe.2024.1390513)
Supplement: Supplementary file 1 [file DataSheet1.pdf]

Supplementary Material

Macroscopic, physical and chemical properties of polyurethane-formulations upon incorporation of Tinuvin 770 DF additive.

1 Rationale

The main function of additives in the polymeric industry is improving the processability of materials or to provide them a specific property of interest. In this work, we incorporated a UV-stabilizer, Tinuvin 770 DF, to produce a lacquer-film with dual functionality: stability to UV-light (to avoid the photodegradation of material) and antimicrobial (to avoid the biodegradation of the material and inhibiting the colonization and growth of potential harmful microorganisms to humans). Specifically, we carried out a comprehensive evaluation to assess both the physical and chemical impact of the incorporation of Tinuvin 770 DF in polyurethane-based formulations (PUR-formulations), as it is essential to ensure the processability of the polymeric formulation, while achieving the desired properties in the final product. Since Tinuvin 770 DF is a powder additive, this evaluation allowed not only the assessment of its dispersion/solubilization in the solvent system being used, but also the analysis of its impact on lacquer viscosity, gloss, and the stability of this additive in the presence of other lacquer constituents.

2 Methodology

The liquid lacquer-formulations were produced and deposited on a polyethylene terephthalate (PET) inert surface to obtain lacquer-films, according to the protocol described in the Material and Methods section. For liquid lacquer-formulations, the runoff time and FTIR analysis was evaluated. For lacquer-films, macroscopic alterations of the films were assessed, as well as FTIR and Raman spectroscopy evaluations, mechanical tests, and gloss value analyses were performed. Control lacquer-formulations (without Tinuvin 770 DF) were prepared, characterized, and used as a reference for comparison.

2.1 Runoff time of lacquer-formulations

Runoff time or flow time can be described as the time it takes for a liquid to start to flow through the orifice of the cup ford until the time the flow breaks (ISO ISO2431, 2019). The runoff time allows to study the resistance and velocity of a fluids (in this study, PUR-based lacquer-formulations) and is an indirect parameter to monitor the viscosity of a fluid. In this work, equal volumes of lacquer-formulations (control and incorporated with Tinuvin 770 DF) were placed on a ford flow cup and the flow tine (in seconds) was measured for each experimental condition and over time. During the experiment, the room temperature was monitored.

2.2 FTIR analysis of lacquer-formulations

In this study, FTIR analyses were done on lacquer-formulations and lacquer-films to assess the impact of incorporating Tinuvin 770 DF on chemical properties of the prepared formulations/films. FTIR-spectra were collected at wavenumber range from 400 to 4000 cm^{-1}, with a resolution of 1 cm^{-1}, over time, in a FT-IR Spectrum Frontier. In addition, FTIR-spectra of Tinuvin 770 DF (powder) were also collected.

2.3 Raman spectroscopy analysis of lacquer-film formulations

To confirm the impact on Tinuvin 770 DF incorporation Raman spectroscopy analysis was done on control lacquer-film and on antimicrobial lacquer-film with 0.5% of Tinuvin 770 DF. Raman spectra were acquired using a confocal Raman microscope (LabRam Soleil from Horiba Scientific), equipped with a green laser (λex = 532 nm) with a maximum output of 75 mW. Spectra were collected using a 50x objective, a laser power of 19 mW (25%), grating 600 (500 nm), and 1s of acquisition time with 10 accumulations, in the range of 400-4000 cm^{-1}.

2.4 Gloss analysis of lacquer-film formulations

To evaluate the optical and surface properties of the prepared lacquer-films, gloss was measured, using a single angle glossmeter with an incidence angle of 60° (Micro-gloss 60° supplied by BYK Instruments). For each experimental condition at least 8 independent reads were recorded in different locations across the surface of the lacquer-film.

2.5 Mechanical evaluation of lacquer-film formulations

Mechanical assays were conducted to assess surface scratch resistance. Prior to testing, the samples were cut into squares measuring 140 x 60 mm and stored for 24 hours at 23±2ºC and 50±6% relative humidity. The assay was performed by subjecting the samples to a force of 3N using an ERICHSEN SmartPen equipped with a 0.75mm diameter needle in a single motion. The samples were observed and photographed in an OLYMPUS SZ61 microscope with an image record system OLYMPUS SC50.

3 Results and Discussion

As represented in Supplementary Figure 1, both control and antimicrobial lacquer-films showed a regular white cast surface, with well-defined borders. As expected, there were no significant differences between these films, indicating that the incorporation of Tinuvin 770 DF did not impact the visual appearance of control lacquer-films.

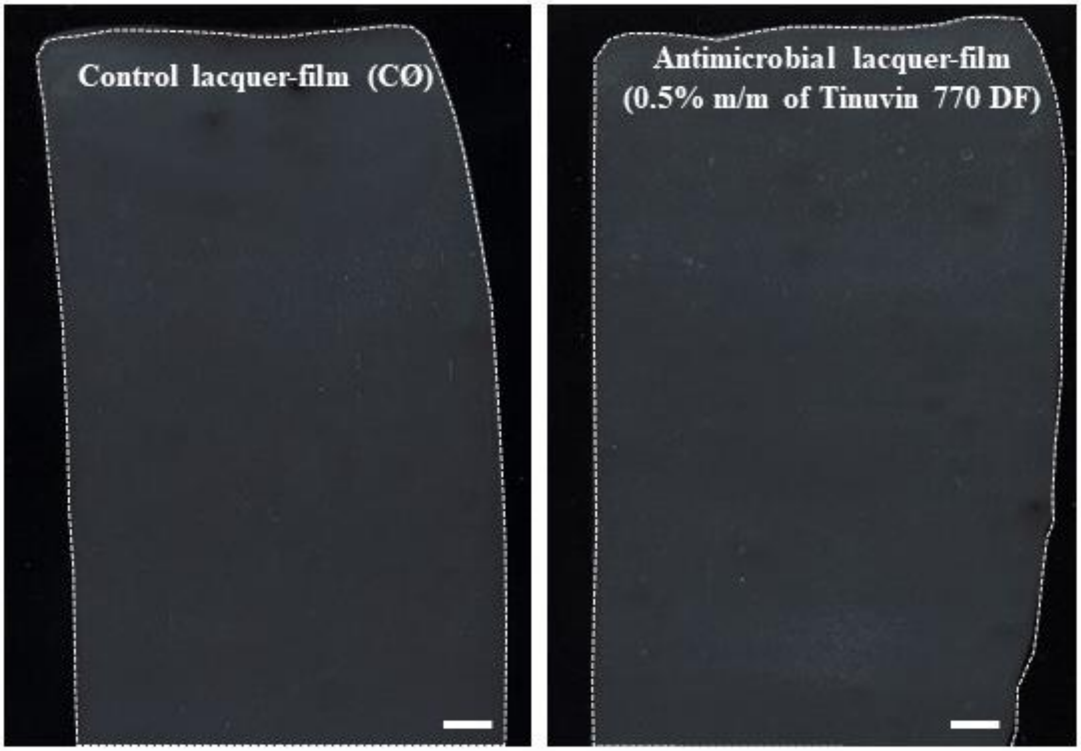

Supplementary Figure 1. Macroscopic view of produced lacquer-films: control lacquer-films (CØ) and antimicrobial lacquer-film (incorporating 0.5% (m/m) of Tinuvin 770 DF). Dashed lines represent the borders of applied lacquer-films on PET substrate. Scale bar = 1 cm.

All lacquer-formulations showed an increase in runoff time, over time, regardless the concentration of Tinuvin 770 DF, in particular after 2 h. Between 0 and 2 h, the behaviour of the lacquer-formulations varied according to the mass concentration of the additives, as depicted in Supplementary Supplementary Figure 2. The control lacquer-formulation without Tinuvin 770 DF, showed a 22 s runoff time from 0 to 8 h, while the lacquer-formulation with 0.5% (m/m) of Tinuvin 770 DF, which represents the original formulation of the antimicrobial lacquer-films used in this work, only revealed an 11 s increase in runoff time. These results are important as they show that the addition of Tinuvin 770 DF does not significantly impact on lacquer viscosity, at least up to 8 h after production.

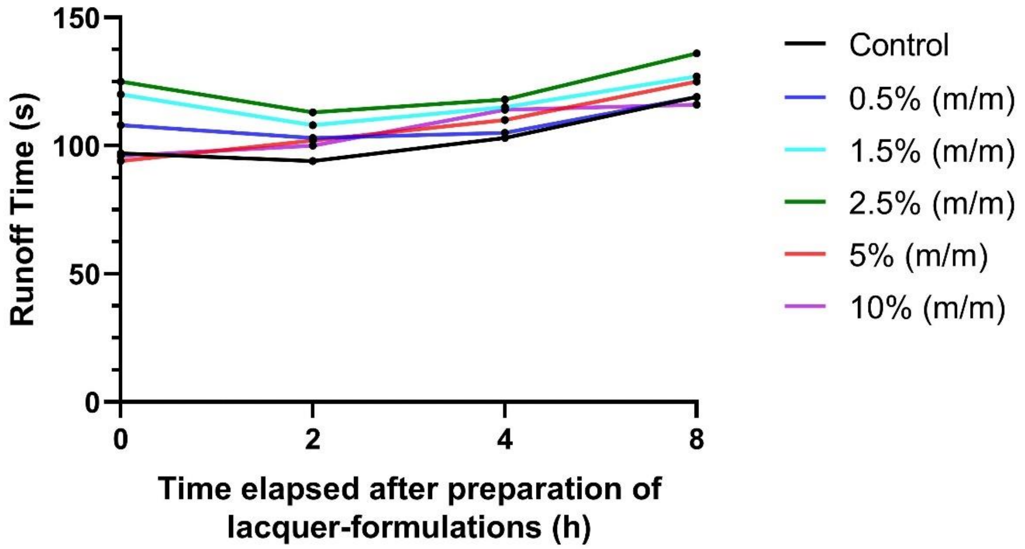

Supplementary Figure 2 – Runoff time of PUR-based lacquer-formulations: Control represents the PUR lacquerformulation without incorporation of Tinuvin 770 DF, and 0.5, 1.5, 2.5, 5 and 10% (m/m) represents the lacquer-formulations with increasing concentrations of Tinuvin 770 DF.

Regarding FTIR analysis no significant differences in the spectrum were observed between control and lacquer-formulations incorporated with Tinuvin 770 DF, regardless the concentration tested over time, at least up to 8 h after production of the lacquers (Supplementary Supplementary Figure 3 and 4). However, for the highest concentrations of Tinuvin 770 DF (5 and 10% (m/m)) there are differences in the FTIR spectrum compared to lower concentrations and control lacquer-films, where additional specific bands between 1100 and 1400 cm^{-1} are detected. This zone is called the 'fingerprint region' and it is frequently used to distinguish specific molecular vibrations, being important to characterize chemical structures (Ramírez-Hernández et al., 2019). In this fingerprint region it is possible to identify specific bands, which were attributed to Tinuvin 770 DF (Supplementary Supplementary Figure 5). These bands are not identified in control lacquer-films as well as in the lacquer-films with lower concentration of Tinuvin 770 DF (<2.55% (m/m)) probably due to the low concentration. Specifically, the following absorption bands were identified: (i) at 1165 cm^{-1}, attributed to -C-O and/or -CH_2 stretching (Yang and Irudayaraj 2000); at 1302 cm^{-1} attributed to -CN stretching (Roychoudhury et al., 2011) and at 1350 cm^{-1} attributed to C-N ring vibration (Pashchevskaya et al., 2010), which are identified by number 1, 4 and 5 in Supplementary Supplementary Figure 5. These bands are observed in spectra of Tinuvin 770 DF and in lacquer-films with highest concentrations of this additive, confirming the incorporation of Tinuvin 770 DF in the lacquer-films. As expected, the intensity of the bands increased with Tinuvin 770 DF content. This occurrence is more evident in band identity at 1350 cm^{-1}.

According to these results is possible to assure that incorporation of Tinuvim 770 at lower concentrations (until 2.5% (m/m)) does not impact the chemical stability of both liquid and solid lacquer-formulations.

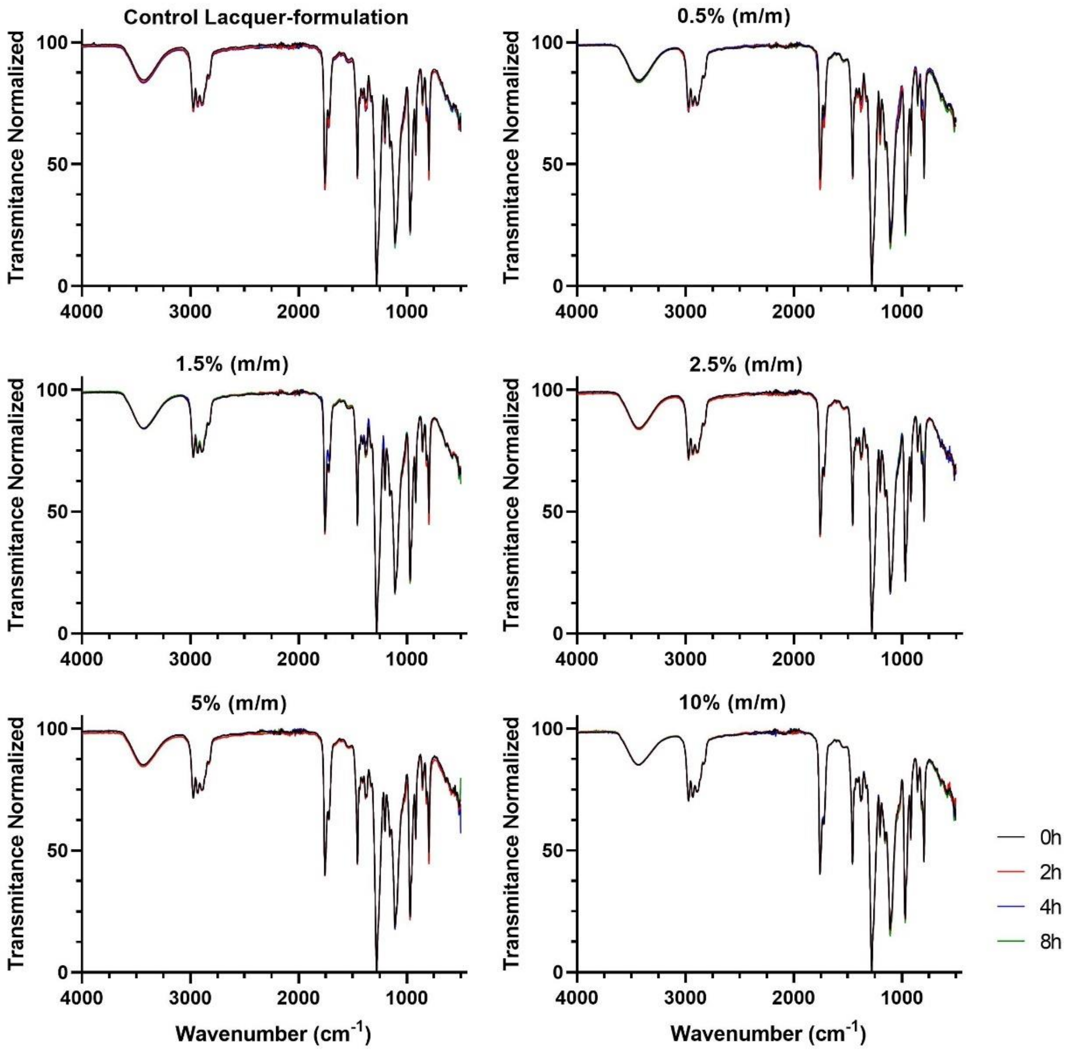

Supplementary Figure 3 – Evolution of FTIR spectra of lacquer-formulations: control and lacquer-formulation incorporated with increasing concentrations of Tinuvin 770 DF (0.5, 1.5, 2.5, 5 and 10% (m/m)), 0, 2, 4 and 8 hours after production.

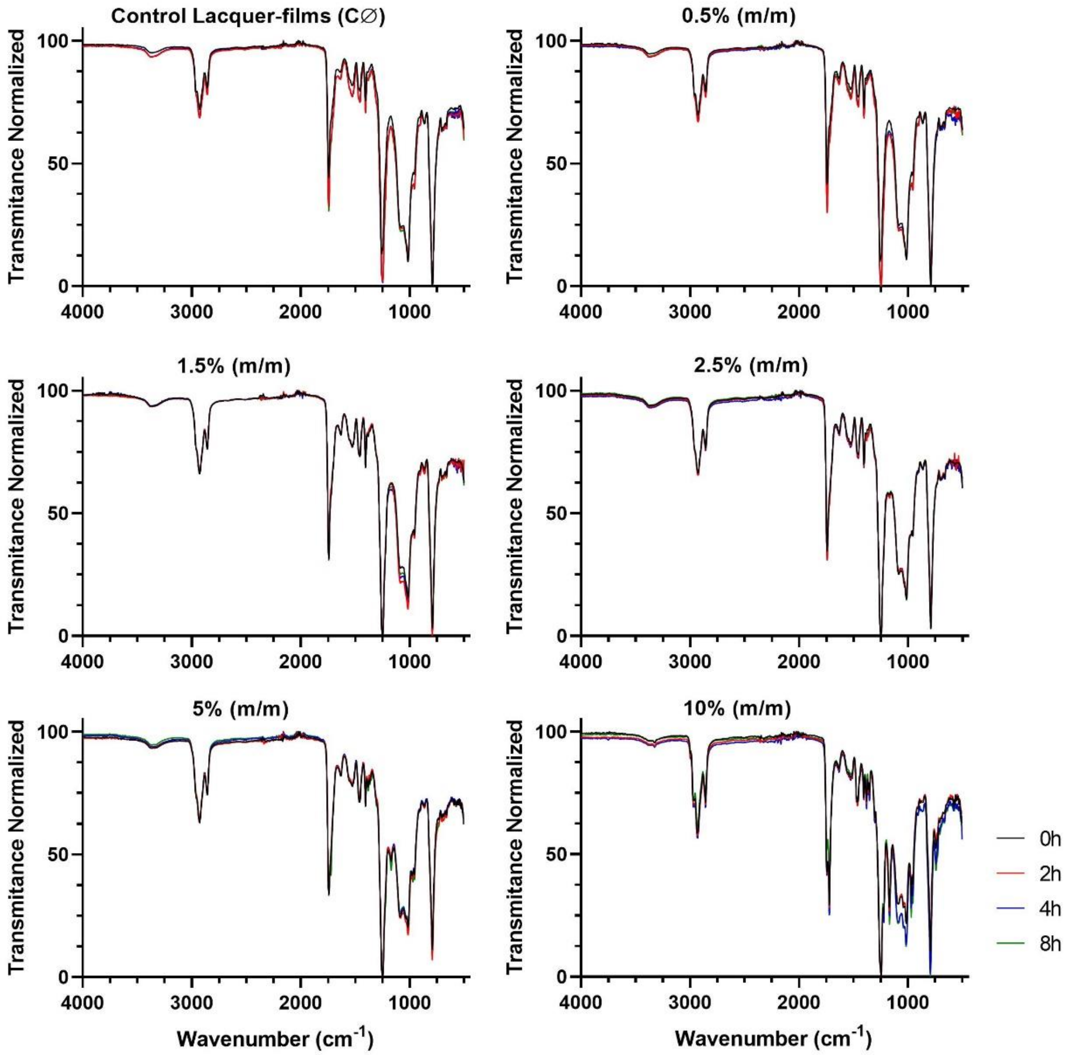

Supplementary Figure 4 – Evolution of FTIR spectra of produced lacquer-films: control and lacquer-films formulated with increasing concentrations of Tinuvin 770 DF (0.5, 1.5, 2.5, 5 and 10% (m/m)), 0, 2, 4 and 8 hours after production.

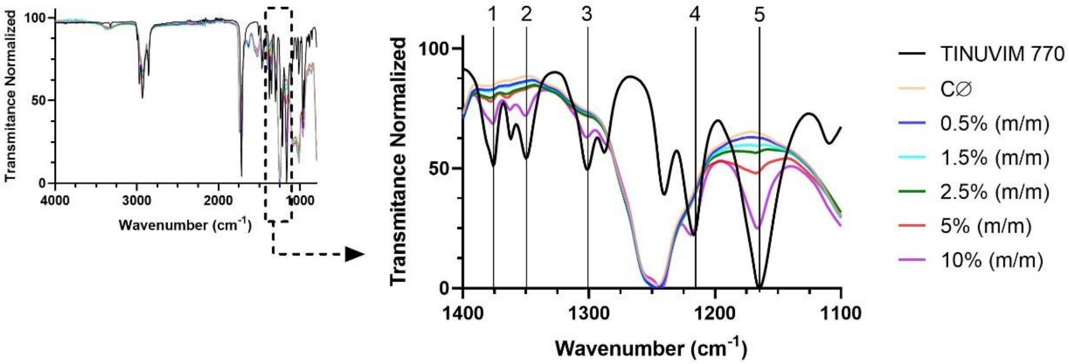

Supplementary Figure 5 – FTIR analysis of lacquer-films produced and Tinuvin 770 DF in pure state (powder): comparison between FTIR spectra of lacquer-films with different concentrations of Tinuvim 770 DF 4 h after production and Tinuvin 770 DF (powder). CØ represents the control lacquer-films (without Tinuvin 770 DF) vs the lacquer-films with increasing concentrations of Tinuvin 770 DF (0.5, 1.5, 2.5, 5 and 10% (m/m)). Numbers represent specific bands used to analyze the FTIR spectrum.

To further confirm the chemical impact of Tinuvin 770 DF incorporation on polyurethane lacquer-films, we conducted Raman spectroscopy analysis on the original formulation of the antimicrobial lacquer-film. Furthermore, surface microscopic images were acquired of tested samples. As illustrated in Supplementary Figure 6A and 6B, a comparable surface morphology was observed between the control and the antimicrobial lacquer-film. Both films, display an irregular and heterogeneous surface, making difficult to identify significant differences between samples, at least at this level. Regarding Raman analysis no significant differences in the spectrum were observed between control and lacquer-formulations incorporated with 0.5% (m/m) Tinuvin 770 DF, as depicted in Supplementary Figure 6C. In fact, is not possible to identify a specific assignments of Tinuvin 770 DF in Raman spectra since they do not have distinctive chemical groups compared to other lacquers components. However, some peaks can have Tinuvin's contribution, specifically the peak at 1733 cm^{-1} (C=O asymmetric stretching) and 790 cm^{-1} (COO deformation) (Iperen et al., 2021). Additionally, some specific bands can be attributed to urethane groups: at 1732 cm^{-1} (urethane amide υ(C=O)); 1303 cm^{-1} (δ(CH) urethane amide) and 1275 cm^{-1} (urethane amide) (Panrell et al., 2003; Durand et al., 2023). It is also possible to observe some peaks attributed to silicon (Si), resulting from the presence of the matting agent in both lacquer-formulations, which is composed, according to the supplier, by polydimethylsiloxane groups. A summary of Raman spectroscopy analysis with bands assignments can be observed in Supplementary Table 2.

Taken together, these results reinforce that the presence of Tinuvin 770 DF at commercial concentration in polyurethane lacquer-formulation does not alter its chemical stability.

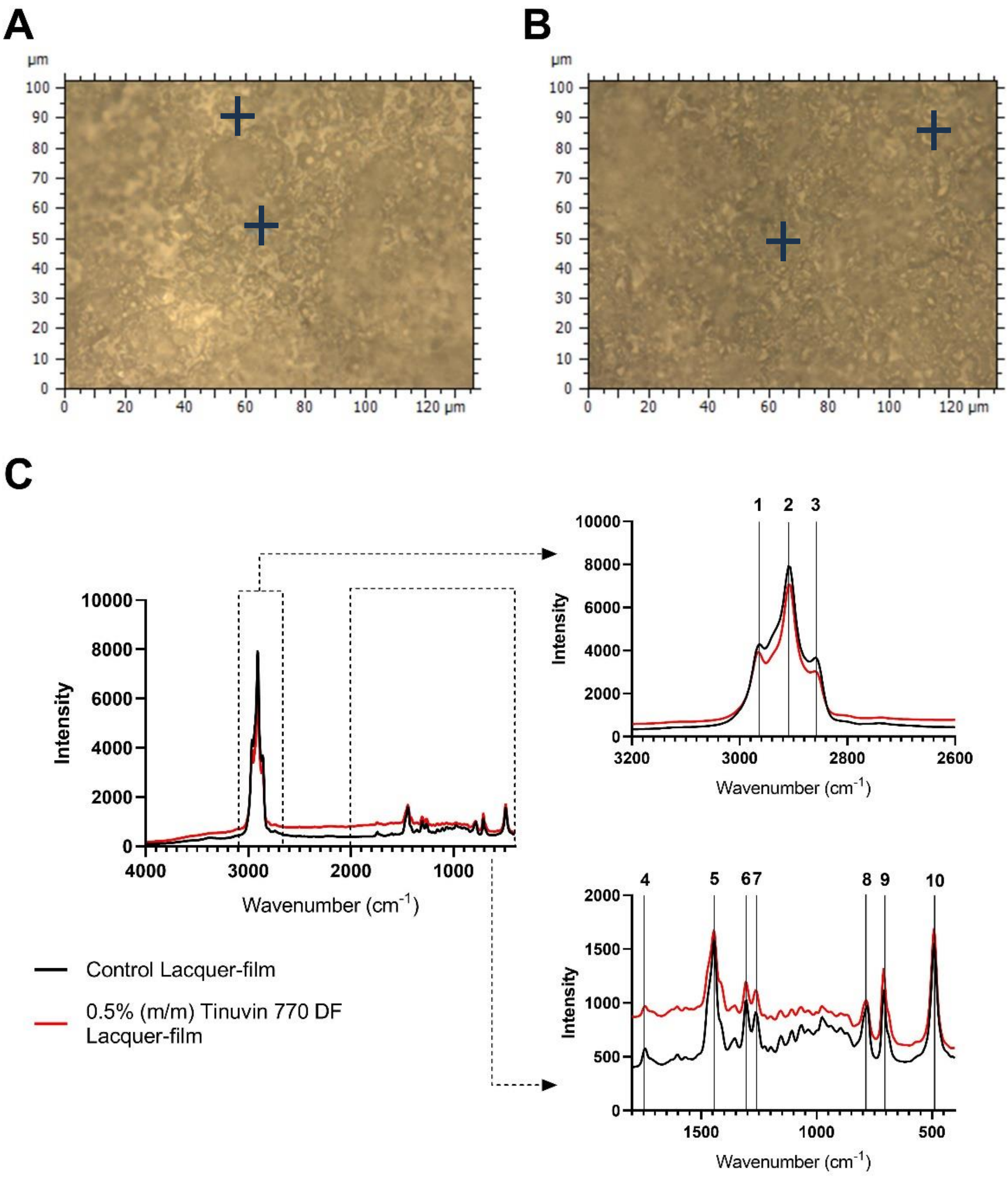

Supplementary Figure 6 – Raman spectroscopy analysis of lacquer-films produced. (A) microscopy surface analysis of control lacquer-film; (B) microscopy surface analysis of lacquer-film incorporated with 0.5% (m/m) of Tinuvin 770 DF, and (C) Raman spectra of control lacquer-film and 0.5% (m/m) of Tinuvin 770 DF lacquer-film. + represents the local of Raman acquisition/analysis. Numbers represent specific bands used to analyze the Raman spectrum.

Supplementary Table 1 – Summary of Raman spectroscopy analysis with bands assignments for control and lacquer-film incorporated with 0.5% (m/m) of Tinuvin 770 DF.

Band	Wavenumber (cm^{-1})	Assignment	Reference
1	2966	C-H sym str/<br>C-H asym str	Jayes et al., 2003
2	2909	C-H sym str	Jayes et al., 2003
3	2858	CH_2 str	Roohpour et al., 2009
4	1732	urethane amide υ(C=O)	Panrell et al., 2003
	1733	Ester (C=O) asym str	Iperen et al., 2021
5	1405	C-O str and O-H deformation	Iperen et al., 2021
6	1303	δ(CH) urethane amide	Panrell et al., 2003<br>Durand et al., 2023
7	1275	urethane amide	Panrell et al., 2003
8	790	COO deformation	Iperen et al., 2021
	790	C-Si-C asym str	Jayes et al., 2003
9	709	C-Si-C, sym str	Jayes et al., 2003
10	490	Si-O, sym str	Jayes et al., 2003

The mechanical assays show that both samples are susceptible to be scratched, as depicted in Supplementary Figure 7. In both samples, the scratch displays a "clean cut" throughout the lacquer-film, without any visible fragments of the lacquer along its path (the visible fragments derived from PET inert surface on which the films were deposited). This could be attributed to good adhesion between the PET surface and the lacquer-film, or to the thickness, which is only a few micrometres. Additionally, is not possible to identify differences between the control and antimicrobial lacquer-film, which indicates that incorporation of 0.5% (m/m) Tinuvin 770 DF in PUR lacquer-films does not change the surface mechanical properties of original lacquer-film.

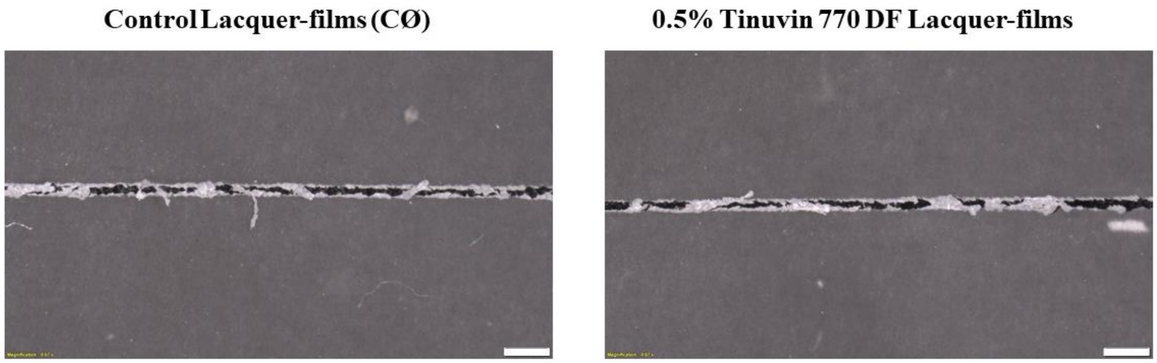

Supplementary Figure 7 – Mechanical evaluation of lacquer-film formulations. Surface scratch resistance for control lacquer-film (left) and for 0.5% (m/m) Tinuvin 770 DF lacquer-film (right). Magnification 0.67x. Scale bar = 1 mm.

The gloss analyses showed that the incorporation of Tinuvin 770 DF can influence the gloss value of the lacquer-films, as presented in Supplementary Table 2. Specifically, the control lacquer-films

showed a gloss value of 6.63 while for lacquer-films with Tinuvin 770 DF above 5% (m/m) of the impact of gloss is a 5-8% reduction in gloss values. The general trend of lower gloss values observed for increasing % (m/m) of additive may be attributed to a matte effect given by the solid particles (white to cream crystalline granules) of Tinuvin 770 DF. Regarding the concentration of Tinuvin 770 DF used in this study and in a commercial context (0.5% (m/m), this did not significantly impact gloss values.

Supplementary Table 2 – Gloss measurements of lacquer-films incorporated with different concentrations of Tinuvin 770 DF.

	Tinuvin 770 DF concentration (% (m/m))*					
	CØ	0.5	1.5	2.5	5	10
Gloss value	6.63±0.11	6.32±0.23	6.41±0.15	6.81±0.24	6.31±0.21	6.11±0.06

***** The values are expressed as the mean±standard deviation, with at least 8 reads in different locations across each film length. CØ represents the control lacquer-films (without Tinuvin 770 DF).

4 Conclusion

The preservation of macroscopic, physical and chemical properties of polymers after incorporation of additives is an important factor in material science and development, given that these additives can modify the functional and structural properties of the final material and ultimately impact their function. In this work, specific macroscopic, physical and chemical properties were tested after the incorporation of Tinuvin 770 DF in a PUR-based formulations. For lacquer-formulations there is no significant changes either in runoff time or in the FTIR and Raman spectra. For lacquer-films only the highest concentrations (5 and 10% (m/m)) impacted the physical and chemical properties. However, these concentrations of Tinuvin 770 DF are not usually used in commercial applications. Typically, UV-stabilizers are used in the range of concentration between 0.05 and 2% (m/m) and the concentration used in the lacquer-films tested in this work contains 0.5% (m/m) of Tinuvin 770 DF.

Collectively, we conclude that the incorporation of 0.5% (m/m) of Tinuvin 770 DF into PUR-based formulations does not impact the macroscopic, physical and chemical properties of the base formulations tested in the framework of this study.

5 References

Duran, S., D’Orlando, A., Mougnard, L., Bourmaud, A., Beaugrand, J. (2023). Combining infrared and Raman spectra to assess MDI localization in novel flax-reinforced automotive composites. *Composites Part C: Open Access*. 12. doi: 10.1016/j.jcomc.2023.100382

Iperen, J., Keulen, H., Keune, K., Abdulah, K., Langh, R. (2021). Crystalline deposits in new display cases at the Rijksmuseum: characterization and Origin. *Studies in Conservation*. 66(5), 253-271. doi: 10.1080/00393630.2020.1811475

ISO2431 (2019). Paints and varnishes — Determination of flow time by use of flow cup. International Organization for Standardization: Switzerland.

Jayes, L., Hard, A. P., Séné, C., Parker, S. F., Jayasoorija, U. A. (2003). Vibrational spectroscopic analysis of silicones: a Fourier Transform-Raman and inelastic neutron scattering investigation. *Anal. Chem*. 75, 742-746. doi: 10.1021/ac026012f

Panrell, S., Min, K., Cakmak, M. (2003). Kinetic studies of polyurethane polymerization with Raman spectroscopy. *Polymer*. 44 (5137-5144). doi: 10.1016/S0032-3861(03)00468-3

Pashchevskaya, N. V., Nazarenko, M. A., Bolotin, S. N., Oflidi, A. I., Panyushkin, V. T. (2010). Effect of the condition of synthesis on the composition and structure of copper(II) complexes with benzimidazole. *Russ. J. Inorg. Chem*. 55, 1425–1432. doi: S0036023610090159

Ramírez-Hernández, A., Aguilar-Flores, C., Aparicio-Saguilán, A. (2019). Fingerprint analysis of FTIR spectra of polymers containing vinyl acetate. *DYNA*. 86, 198–205. doi: 10.15446/dyna.v86n209.77513

Roohpour, N., Wasikiewicz, J. M., Moshaverinia, A., Paul, D., Rehman, I. U., Vadgama. P. (2009). Isopropyl myristate-modified polyether-urethane coatings as protective barriers for implantable medical devices. *Materials*. 2, 719-733. doi:10.3390/ma2030719

Roychoudhury, M., Gaurav, P. K., Manohar, R., Prajapati, A. K. (2011). Analysis of mesogenic characteristics of 6-chloro-benzothiazol-2-yl-(4-hexadecyloxyphenyl) diazene-a smectic liquid crystal. *Mol. Cryst. Liq. Cryst*. 537, 141–154. doi: 10.1080/15421406.2011.556472

Yang, H., Irudayaraj, J. (2000). Characterization of semisolid fats and edible oils by Fourier Transform Infrared Photoacoustic spectroscopy. *JAOCS*. 77, 291–295. doi: 10.1007/s11746-000-0048-y
